# Native edaphoclimatic regions shape soil communities of crop wild progenitors

**DOI:** 10.1093/ismeco/ycaf143

**Published:** 2025-09-24

**Authors:** María José Fernández-Alonso, Miguel de Celis, Ignacio Belda, Javier Palomino, Carlos García, Juan Gaitán, Jun-Tao Wang, Luis Abdala-Roberts, Fernando D Alfaro, Diego F Angulo-Pérez, Manoj-Kumar Arthikala, Danteswari Chalasani, Jason Corwin, Duan Gui-Lan, Antonio Hernandez-Lopez, Kalpana Nanjareddy, Siddaiah Chandra Nayaka, Babak Pasari, Thanuku Samuel Sampath Kumar Patro, Appa Rao Podile, Teresa Quijano-Medina, Daniela S Rivera, Pullabhotla Venkata Subba Rama Narshima Sarma, Salar Shaaf, Pankaj Trivedi, Qingwen Yang, Yue Yin, Eli Zaady, Yong-Guan Zhu, Brajesh K Singh, Manuel Delgado-Baquerizo, Pablo García-Palacios, Ruben Milla

**Affiliations:** Area of Biodiversity and Conservation, Department of Biology and Geology, Physics and Inorganic Chemistry, Rey Juan Carlos University, C/ Tulipán s/n, 28933 Móstoles, Spain; Departamento de Geología y Geoquímica, Facultad de Ciencias, Universidad Autónoma de Madrid, 28049 Madrid, Spain; Departamento de Suelo, Planta y Calidad Ambiental, Instituto de Ciencias Agrarias, Consejo Superior de Investigaciones Científicas, 28006 Madrid, Spain; Department of Genetics, Physiology and Microbiology, Faculty of Biology, Complutense University of Madrid, 28040 Madrid, Spain; Area of Biodiversity and Conservation, Department of Biology and Geology, Physics and Inorganic Chemistry, Rey Juan Carlos University, C/ Tulipán s/n, 28933 Móstoles, Spain; Department of Soil and Water Conservation and Organic Waste Management, CEBAS-CSIC, 30100 Murcia, Spain; Instituto de Suelos - INTA Castelar, CONICET, Universidad Nacional de Luján, 6700 Luján, Buenos Aires, Argentina; Hawkesbury Institute for the Environment, Western Sydney University, Penrith, NSW 2751, Australia; Global Centre for Land-Based Innovation, Western Sydney University, Penrith, NSW 2751, Australia; School of Science, Western Sydney University, Penrith, NSW 2751, Australia; Departamento de Ecología Tropical, Campus de Ciencias Biológicas y Agropecuarias, Universidad Autónoma de Yucatán, 97000 Mérida, Yucatán, Mexico; GEMA Center for Genomics, Ecology and Environment, Universidad Mayor, Santiago 8580745, Chile; Unidad de Recursos Naturales, Centro de Investigación Científica de Yucatán, 97205 Mérida, Yucatán, Mexico; Ciencias Agrogenómicas, Escuela Nacional de Estudios Superiores Unidad León, Universidad Nacional Autónoma de México, León 37689, Guanajuato, Mexico; Department of Plant Sciences, School of Life Science, University of Hyderabad, Hyderabad, 500046, Telangana, India; Microbiome Network and Department of Agricultural Biology, Colorado State University, Fort Collins, CO 80523-1177, United States; State Key Laboratory of Urban and Regional Ecology, Research Center for Eco-Environmental Sciences, Chinese Academy of Sciences, Beijing 100085, China; Ciencias Agrogenómicas, Escuela Nacional de Estudios Superiores Unidad León, Universidad Nacional Autónoma de México, León 37689, Guanajuato, Mexico; Ciencias Agrogenómicas, Escuela Nacional de Estudios Superiores Unidad León, Universidad Nacional Autónoma de México, León 37689, Guanajuato, Mexico; Department of Studies in Biotechnology, University of Mysore, Manasagangotri, Mysuru 570006, Karnataka, India; Department of Agronomy and Plant Breeding, Islamic Azad University, Sanandaj Branch, Sanandaj 6616935391, Iran; Agricultural Research Station (ACRIP center-small millets), Acharya NG Ranga Agricultural University, Vizianagaram 535001, Andhra Pradesh, India; Department of Plant Sciences, School of Life Science, University of Hyderabad, Hyderabad, 500046, Telangana, India; Departamento de Ecología Tropical, Campus de Ciencias Biológicas y Agropecuarias, Universidad Autónoma de Yucatán, 97000 Mérida, Yucatán, Mexico; GEMA Center for Genomics, Ecology and Environment, Universidad Mayor, Santiago 8580745, Chile; Department of Plant Sciences, School of Life Science, University of Hyderabad, Hyderabad, 500046, Telangana, India; Leibniz Institute of Plant Genetics and Crop Plant Research (IPK), 06466 Seeland, Germany; Microbiome Network and Department of Agricultural Biology, Colorado State University, Fort Collins, CO 80523-1177, United States; National Key Facility for Crop Gene Resources and Genetic Improvement, Institute of Crop Sciences, Chinese Academy of Agricultural Sciences, Beijing 100081, China; State Key Laboratory of Urban and Regional Ecology, Research Center for Eco-Environmental Sciences, Chinese Academy of Sciences, Beijing 100085, China; Laboratorio de Biodiversidad y Funcionamiento Ecosistémico, Instituto de Recursos Naturales y Agrobiología de Sevilla (IRNAS), CSIC, 41012 Sevilla, Spain; Katif Research & Development Center, Sdot Negev, Netivot 8771002, Israel; State Key Laboratory of Urban and Regional Ecology, Research Center for Eco-Environmental Sciences, Chinese Academy of Sciences, Beijing 100085, China; Hawkesbury Institute for the Environment, Western Sydney University, Penrith, NSW 2751, Australia; Global Centre for Land-Based Innovation, Western Sydney University, Penrith, NSW 2751, Australia; Laboratorio de Biodiversidad y Funcionamiento Ecosistémico, Instituto de Recursos Naturales y Agrobiología de Sevilla (IRNAS), CSIC, 41012 Sevilla, Spain; Departamento de Suelo, Planta y Calidad Ambiental, Instituto de Ciencias Agrarias, Consejo Superior de Investigaciones Científicas, 28006 Madrid, Spain; Department of Plant and Microbial Biology, University of Zurich, CH-8008 Zurich, Switzerland; Area of Biodiversity and Conservation, Department of Biology and Geology, Physics and Inorganic Chemistry, Rey Juan Carlos University, C/ Tulipán s/n, 28933 Móstoles, Spain; Global Change Research Institute, Rey Juan Carlos University, 28933 Móstoles, Spain

**Keywords:** centres of origin, crop wild progenitors, ecoregions, edaphoclimate, rewilding, soil core microbiome, soil core microfauna

## Abstract

Unveiling the soil biological communities ecologically associated with crop wild progenitors (CWPs) in their habitats of origin is essential for advancing productive and sustainable agriculture. A field survey was conducted to investigate the edaphoclimatic conditions and soil bacterial, fungal, protist, and invertebrate communities of 125 populations of direct progenitors of major crops for world agriculture. The wild populations clustered into four ecoregions shaped by two edaphoclimatic dimensions: one summarizing variations in soil sand contents and nutrients concentrations, and the other featuring changes in aridity, soil pH, and carbon storage potential. We identified a common soil core community across CWPs that varied significantly along deserts to tropical seasonal forests and savannas. The assembly of the soil core community was driven by varying environmental preferences amongst soil biodiversity kingdoms, reflecting potential shifts in their functional profiles. The tropical ecoregion exhibited higher proportion of acidophilic bacteria, fungal, and protist parasites, whilst desert ecosystems harboured greater abundances of saprophytic fungi and heterotrophic protists. Moreover, CWPs displayed unique microhabitats that incorporate variability into the soil community assembly. Our work reveals the biogeography of soil communities associated with CWPs, the first step towards the development of microbial rewilding initiatives.

## Introduction

The global food system relies on a small set of crops that trace their origins to specific geographic regions around the world [1]. In these native habitats, the crop wild progenitors (CWPs) and their associated soil microbiomes have historically co-evolved, establishing interactions that influence plant performance and ecosystem functioning [2]. Hereby, the soil microbiome associated with the wild progenitors of major crops is an untapped resource to meet global agricultural challenges [3, 4]. However, today, we lack the most basic understanding on the soil microbiome at the crop centres of origin, especially when considering multiple domestication regions and crops simultaneously [5]. The processes of plant domestication and farming have altered host–microbial relationships in modern crops [6], undermining the functionality, resilience, and sustainability of croplands [7]. Reinstating part of the ancient biotas of CWPs that support beneficial interactions in modern crops constitutes the foundations of the microbiome rewilding framework [8]. For this to happen, assessing the environmental preferences of soil biodiversity at the habitats of origin of major crops is a first step.

Soils are the most biodiverse habitat on Earth [9], and are the principal inoculum source of microbes and invertebrates colonizing the rhizosphere and root tissues [10, 11]. Soil and climatic factors are of paramount importance in determining soil community assembly and further host plant control on microbiome recruitment [12]. However, most studies addressing the microbiome of CWPs do not focus on native soils under the environmental conditions in which wild populations thrive [6, 13]. Notably, one study revealed that certain taxa present in native soils of a CWP were absent in cropland soils, with the latter exhibiting increased diversity but a simpler network structure, suggesting a homogenized and less specialized soil microbiome [14]. Microbial taxa associated with CWPs may display traits linked to local conditions and stressors in their habitats of origin that are absent in modern agricultural soils [8, 14]. Hence, to take advantage of the microbiomes of CWPs to improve current crops, we must understand their distribution and ecology in order to develop a baseline for more applied research. However, a comprehensive biogeographical investigation of soil biodiversity patterns across multiple kingdoms is absent. This approach can identify key members of the soil microbiota of CWPs to be reinstated using microbial inoculants and synthetic communities that help current crops cope with changing environmental conditions [7].

The joint characterization of the edaphoclimatic context and the associated soil biota amongst CWPs allows the establishment of regions with interrelatedness ecological components. It is particularly meaningful to identify sets of soil biotic taxa that most effectively colonize the native environments and thereby are dominant, and also being associated with multiple CWPs, suggesting host–microbe relationships with important functional roles [15]. This ecoregion perspective can assist in identifying which components of the soil core microbiome of CWPs could be most effectively and successfully transferred to modern agroecosystems [16, 17]. Additionally, environmental characteristics at the sites of origin of CWPs may provide underlying factors modulating the evolution and breeding of modern crop phenotypes and, consequently, agroecosystem functioning [18]. Yet, the ecological legacy of modern crops from their sites of origin is typically overlooked in agricultural studies [19]. Investigating in which ecoregions CWPs inhabit and how they relate with the associated soil core community may offer a novel framework for better explaining the effects of domestication on plant phenotypes, and for microbial rewilding studies in agriculture.

**Figure 1:**
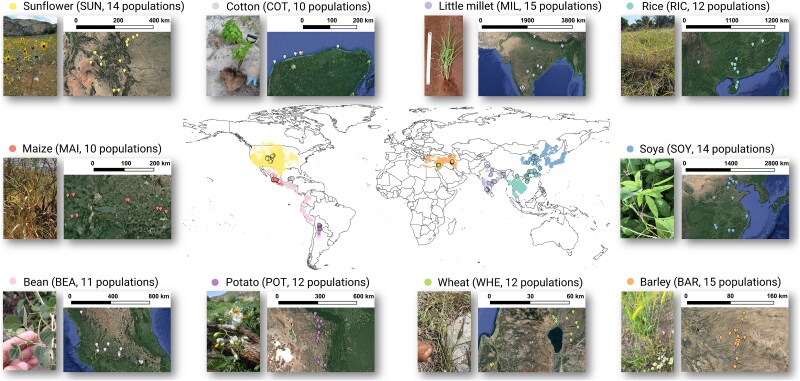
Global distribution of the wild progenitors of the 10 selected crops. Distributions are denoted as borderless points, whilst the specific populations surveyed in this study are bordered points. Global occurrences were retrieved from the global biodiversity information facility database [20]. The common names of modern crops designate the wild populations surveyed in this project. The abbreviations of the crop names, and the total number of surveyed populations are given in brackets. The wild progenitors of the crops are: *Gossypium hirsutum* L., cotton; *Glycine max* subsp. *soja* (Siebold & Zucc) H. Ohashi, soja; *Helianthus annuus* L., sunflower; *Hordeum vulgare* subsp. *spontaneum* (K. Koch) Asch. & Graebn., barley; *Oryza rufipogon* Griff., rice; *Panicum sumatrense* Roth, little millet; *Phaseolus vulgaris* L., common bean; *Solanum berthaultii* Hawkes, potato; *Triticum dicoccoides* (Körnicke) G. Schweinfurth, wheat; *Zea mays* subsp. *parviglumis* Iltis & Doebley, maize. Images of wild populations taken during field campaigns. Landsat images from Google earth pro (https://earth.google.com/web). Sunflower image retrieved from [58].

Here, we investigated the biogeography and edaphoclimatic factors associated with the soil core microbiome and micro-fauna at the habitats of origin of major crops. We performed a global standardized field survey at 125 wild populations across the distribution ranges of the recognized CWPs of 10 major crops (maize, rice, wheat, potato, bean, little millet, barley, sunflower, soya, and cotton; Fig. 1), which account for 40% of the total area annually harvested worldwide and 61% of the global production of primary crops in the period 2012–21 [20, 21]. Our survey compiles information on climate, potential ecosystem carbon sinks, soil parameters, and the core taxa and co-occurrence networks of soil bacteria, fungi, protists, and invertebrates from six domestication centres (North America, Mesoamerica, Central Andes, Fertile Crescent, Indomalaya region, Yangtze and Yellow River valleys). We addressed the following questions: (i) what is the edaphoclimatic niche of CWPs in their native habitats?, (ii) is there a common soil core community (microbes and microfauna) embracing different CWPs and ecoregions?, and (iii) which edaphoclimatic factors determine the soil biodiversity of CWPs?

## Materials and methods

To investigate the soil biogeography at the habitats of origin of major crops, we sampled 125 wild populations of 10 species of CWPs that encompassed broad geographic and environmental gradients. For each population, we gathered climate, potential ecosystem carbon sinks (i.e., energy availability to trophic networks), soil fertility, and physiochemical data. We measured soil bacteria, fungi, protist, and invertebrate communities through amplicon sequencing. We used principal component and module analyses to characterize the sites of origin of CWPs by distinct edaphoclimatic features (i.e., ecoregions). Then, we identified the core members of ancestral agriculture using abundance (10% more abundant phylotypes) and prevalence (phylotypes present in the populations of at least four CWPs) criteria. We calculated co-occurrence networks to identify the main modules of the soil biotic community and assessed the relative abundance patterns across ecoregions. Finally, we used linear mixed effects models to identify the environmental drivers responsible for clustering patterns of the soil core members of ancestral agriculture.

### Crop wild progenitors

We selected 10 major crops of global relevance (maize, rice, wheat, potato, bean, little millet, barley, sunflower, soya, and cotton), including a woody crop, a tuberous crop, and several herbaceous crops harvested for their seeds. We used the Crop Origins and Phylo Food database to identify crops´ geographic origin and their most likely wild progenitor (https://rubenmilla.github.io/Crop_Origins_Phylo/, [22]). For each CWP, we selected 10–15 truly wild populations in habitats across its native range of distribution, maximizing the environmental variation amongst populations (altitudinal/latitudinal or other edaphoclimatic gradients). In each population, we surveyed a 20 m × 20 m plot. The mean separation distance between native populations within each species was 364 km (ranging from 1 to 2265 km). In total, we sampled 125 populations of CWPs across disparate regions of the world (Fig. 1).

### Soil sampling

In 2020 and 2021, we collected native soil samples from each wild population, focusing on soil communities associated with CWPs. This soil compartment is widely used in ecological and microbiome studies [23], harbours the highest biodiversity and stands as the foremost source of plant microbes [24]. We selected five plants each spaced at least 5 m apart during the flowering stage, which is a period that facilitates the identification of CWPs, guarantees their active growing, and is characterized by key plant–soil interactions [24]. We uprooted the plants along with an entire soil monolith within a 10-cm radius and to a depth of 20-cm. We collected the soil that was not intimately attached to the plant roots by shaking off any soil not tightly adhering to them [23]. We combined soil samples from each plot to create a composite and homogeneous sample for each population. We immediately sieved the soil samples at 2 mm and stored them at 4°C until shipment to the Universidad Rey Juan Carlos (URJC, Madrid, Spain) for processing and analysis. Once in Spain, we froze a subset of each soil sample at −20°C until DNA extraction. We air dried and stored the remaining of each sample for physiochemical analyses. Little millet samples could not leave the country due to legal constraints and they were processed at the University of Hyderabad (Telangana, India) and extracted for soil DNA. Then, soil physiochemical properties were analysed at the International Crops Research institute for the Semi-arid tropics (ICRISAT, Hyderabad, India), following the same procedures applied to the rest of the CWPs.

### Soil physiochemical analyses

We analysed electrical conductivity and pH in deionized water extracts (suspension ratio of 1:5 and 1:2.5 weight:volume, respectively). We estimated soil texture (percentage of sand) using the Bouyoucos hydrometer method [25]. We determined the total soil carbon and nitrogen concentrations by the Dumas dry combustion procedure using an elemental analyser (C/N Flash EA 112 Series-Leco Truspec). We determined the soil organic carbon (SOC) by dry combustion in 2 N HCl pre-treated samples, except for soil samples of little millet that were analysed by the Walkley–Black method. We converted SOC data obtained with the Walkley–Black to dry combustion SOC using an established conversion factor of 1.15 [26]. We extracted total concentrations of soil micronutrients (Ca, Na, K, B, Mg, Mn, Cu, Fe, and Zn) using wet acid digestion and determined them using inductively coupled plasma optical emission spectrometry (ICAP 6500 DUO ICP-OES spectrometer, Thermo-Scientific) according to ISO 11885:1996. We analysed mineral nitrogen and available phosphorus (in the form of NH_4_^+^, NO_3_^−^ and PO_4_^−^) in soil water extracts by ionic exchange chromatography with a cation-exchange column (Metrosep A Supp 5–250/4.0, Herisau).

### Amplicon sequencing

We extracted soil DNA from 0.5 g of a sample using the DNeasy PowerSoil Kit (Qiagen) according to the manufacturer’s instructions. We checked the quality and quantity of DNA using a NanoDrop 2000 (Thermo Fisher Scientific, USA) and Qubit Fluorometer (Thermo Fisher Scientific, USA). We determine the biotic community profiling through amplicon sequencing on the Illumina MiSeq platform of the Next-Generation Sequencing Facility at the Western Sydney University (Richmond, NSW, Australia). For the library preparation, we used the primer set 799F /1193R to amplify the bacterial 16S rRNA gene (V5-V7 region), which effectively reduces the amplification of host DNA (e.g. chloroplasts and other organellar sequences) and improves bacterial community detection [27, 28]. The eukaryotic 18S rRNA gene Euk1391f/EukBr was sequenced to study both protist and invertebrates [29]. We used the primer sets FITS7/ITS4 to amplify the nuclear ribosomal internal transcriber spacer 2 (ITS2 region), which is a common maker for fungal communities [30].

We trimmed primers from paired raw reads and performed an end-trim (base quality <26) to increase the rate of successful merge. We merged the resultant paired reads using USEARCH [31] and FLASH [32] and discarded sequences of low quality (maximum expected error of 1.0). We denoised the sequences (error-correction) and clustered them into zOTUs (100% sequence similarity) using UNOISE3 [33]. We annotated representative sequences against the UNITE database (2022.05) using QIIME2 [34] for ITS data, and the SILVA database for 16S and 18S rDNA data, respectively. We rarefied at 12000, 8000, 5000, and 250 reads per sample for bacteria, fungi, protists, and invertebrates, respectively (Fig. S1). We explored the potential lifestyle of the soil fungal community using the FungalTraits v1.2 database based on the fungal ITS data [35].

### Environmental data

We gathered climate and ecosystem productivity data, for the coordinates of each population, from global databases. We obtained climatic variables [mean annual precipitation, mean annual temperature (MAT), precipitation seasonality (PSEA), temperature seasonality, and mean diurnal range temperature (MDR)] from the WorldClim dataset version 2 [36], and the UNEP aridity index (AI = precipitation/potential evapotranspiration, where the higher the index the lower the aridity) from the Global Aridity Index and Potential Evapotranspiration Climate Database version 2 [37]. We used the Normalized Difference Vegetation Index (NDVI) as a proxy for net aboveground primary productivity, based on values from the MODIS satellite imagery MOD13Q1. We followed a standard procedure for NDVI calculation at a global scale. We acquired 23 NDVI values per year with a pixel size of 250 × 250 m and used them to calculate annual NDVI data for each year in the period from 2000 to 2021. To do so, we averaged the product values between the date of the minimum NDVI ($n$) and the date $n-1$ of the following year at each site. MODIS data are geometrically and atmospherically corrected and include a reliability index of data quality based on the environmental conditions in which the data were recorded.

### Data analyses

We carried out exploratory data analysis to inspect the structure of missing values (4% of the environmental database). We imputed the multivariate missing data using the *mice* package and the predictive mean matching model with 999 iterations [38]. Data imputation did not alter the distribution of original data as shown in density plots (Fig. S2).

### Edaphoclimatic distribution of CWPs

To outline the bioclimatic characterization of the populations of the CWPs surveyed, we used Whittaker’s biomes classification [39], the aridity index and NDVI (an indirect measure of vegetation cover and primary productivity). To evaluate the edaphoclimatic differences amongst the populations surveyed, we ran a Principal Component Analysis (PCA) on 18 centred and scaled local soil and climate variables. The variables with the highest square cosine were used to interpret the PCA axes (*FactoMineR* package [40], Fig. S3). To describe ecoregions with similar edaphoclimatic conditions, we performed a module analysis based on the first 5 PCA axes, which captured 74% of the total variance. We multiplied the PCA axes by the square root of their eigenvalues to weight the classification outcome according to their importance. We calculated Euclidean distances to overcome the multicollinearity of the edaphoclimatic variables and exploit their orthogonal properties. We used the K-means algorithm and the Calinski–Harabasz index to select the optimal number of groups (“cascadeKM function”, *vegan* package [41], Fig. S4). Clustering results were represented using elliptic shapes based on multivariate t-distribution. To test the differences in the dimensions of the edaphoclimatic space by CWPs, we used the Wilcoxon signed-rank test and linear models with the applied Bonferroni-estimated marginal mean comparisons as post-hoc analyses.

### Assignment of soil core community

Given our global focus, we identified the soil core microbiome and microfauna of ancestral agriculture based on both relevance and prevalence criteria [15]. First, we kept only the 10% most common phylotypes in terms of average relative abundance. Then, within that subset of commonest phylotypes, we selected phylotypes present in at least four CWPs (i.e., 40% of the plant species surveyed). We conducted these analyses independently for bacteria, fungi, protists, and invertebrates. The resulting abundance tables contain phylotypes that (i) make a substantial contribution to ecosystem functionality, (ii) are prevalent and thereby adapted to contrasting climates and soils, thus likely providing considerable versatility for their agricultural application, and (iii) reduce potential spurious associations of rare taxa, since zero-inflated databases may confound ecological interpretations in downstream co-occurrence network analyses [42–44]. To enable the identification of overlap and uniqueness patterns of phylotypes in the soil core community across CWPs, we used Upset plots (*ComplexHeatmap* package [45]).

### Structure of the community: co-occurrence network analyses

To explore the structure of the soil core community of ancestral agriculture, we conducted co-occurrence network analyses. We calculated potential co-occurrences between core phylotypes using an improved version of the function originally described in de Celis *et al*. [46], which applies a probabilistic model to presence–absence matrices [47]. This approach yielded a list of phylotypes pairs that co-occurred more frequently than expected by chance. We built independent networks for bacteria, fungi, protists, and invertebrates, where nodes were phylotypes and edges were significant pairwise co-presence between nodes (*P*-value <.05). We determined modularity and clusters (modules of dense connections that are sparsely connected to other areas in the network) using a community finding algorithm via short random walks (“module_walktrap function” from *igraph* package [48]). The modules can indicate soil microorganisms that potentially interact or share environmental preferences, although other mechanisms responsible for community assemblies such as dispersion, diversification, and drift could also underlie the network structure. To investigate whether biogeographical patterns exist in the overall network structures, we computed the proportion of nodes of a given module that were present in wild populations of CWP in each ecoregion (module completeness). Given the unequal number of phylotypes across biotic modules (Table S1), we calculated the standardized relative abundance [z-score, ${z}_{ij}$ in Equation (1)] of phylotypes and averaged them per module and wild population. This standardization allows to obtain a balanced contribution of the phylotypes (i.e. nodes) to the relative abundances of the modules in the networks for downstream analyses.


(1) \begin{equation*} {z}_{ij}=\frac{\left({x}_{ij}-\bar{x}_i\right)}{\sigma_i} \end{equation*}

Where ${z}_{ij}$ represents the number of reads for the phylotype $j$ in the wild population $i$, $\bar{x}_i$ is the average number of reads for wild population $i$, and ${\sigma}_i$ is the standard deviation of the reads for the wild population $i$. To test for the differences in the relative abundance of each module across CWPs, we fit linear models and applied Bonferroni-estimated marginal mean comparisons as *post hoc* analyses.

### Environmental factors determining modules of soil core members

To test if and how the modules in the resulting networks were influenced by environmental factors, we used linear mixed models (LMMs), four groups of predictors (climate: MAT, MDR, PSEA, AI; soil properties: pH, conductivity, sand; soil micronutrient concentrations: NH_4_^+^, NO_3_^−^, PO_4_^−^, Ca, B, K, Mg, Zn, Fe, Mn, Mo; and potential ecosystem carbon sink: mean NDVI, SOC), and the relative abundance (z-score) of the modules as the dependent variable. We retained the above-mentioned subset of variables after checking for multicollinearity patterns in our dataset of environmental predictors by means of the variance inflation factor and using a threshold level of five (*regclass* package, [49]). To deal with the hierarchical design of the survey, we used the identity of the CWPs as a categorical random intercept in the model. The random factor accounts for local drivers affecting microbial community assembly that were not measured and that may be directly attributed to the identity of the CWP species. We optimized the structure of the fixed components in the LMMs through a backward selection procedure of the predictor variables according to the Akaike Information Criterion (*mass* package [50]). Model coefficients were estimated using the restricted maximum likelihood criterion (*lme4* package [51]) and 95% bootstrap confidence intervals were calculated based on 1000 simulations (*jtools* package [52]). To interpret the outcome of the LMMs, we standardized the predictors and decomposed the marginal coefficient of determination (Rm^2^) into unique and shared variance by each group of fixed predictors (*glmm.hp* package [53]). We checked LMM assumptions visually and using the Anderson–Darling normality test. All statistical analyses and visualizations using R version 4.2.4.

## Results and discussion

Our standardized field survey provides the first joint assessment of the soil core community—including bacterial, fungal, protist, and invertebrate phylotypes—and edaphoclimatic components of the most relevant CWPs investigated across their original distribution worldwide. The wild populations of our CWPs clustered into four ecoregions that differed widely in their edaphoclimatic attributes, such as water and energy supply, soil pH, texture, and fertility. Environmental variation amongst ecoregions suggests that the ecophysiological traits of CWPs are likely to differ amongst the more oligotrophic coastal shrubland ecoregion, deserts, and tropical seasonal forests and savannas. We identified the soil core community common to all CWPs, which were dominated by the major soil phyla commonly found in studies surveying soil ecosystems worldwide. Despite this commonality, we also found distinct ecological groups of soil core phylotypes that showed variation in their prevalence across ecoregions, driven by varying environmental preferences amongst kingdoms. These biogeographical patterns of the soil core microbes and microfauna point to changes in their life history strategies, showing increased proportional abundances of acidophilic bacteria, as well as fungal and protist parasites in the tropical seasonal forest and savanna ecoregion. Wild populations displayed specific microhabitats within ecoregions that could play a selective role in the assembly of the soil core microbiome. These findings improve our understanding of the ecology of CWPs and their associated soil communities, which holds significant implications for developing strategies for rewilding in agriculture.

### Ecoregions of crop wild progenitors

**Figure 2:**
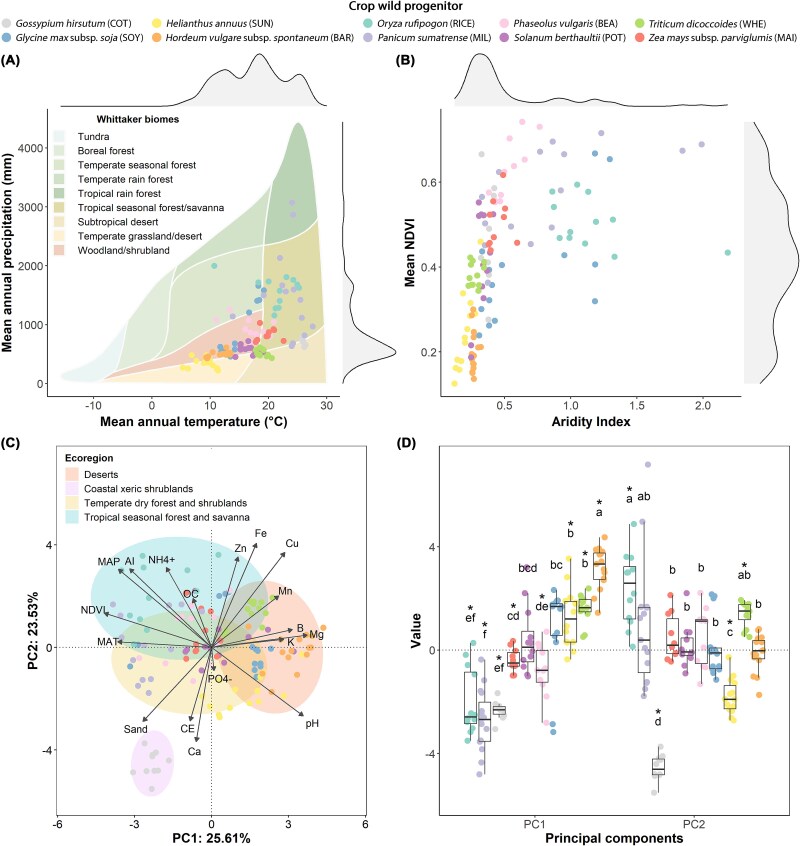
Edaphoclimatic characterization of the populations of crop wild progenitors (CWPs). Coloured circles are the surveyed populations of each CWP, and abbreviations in brackets indicate their corresponding modern crop. (A) Climate distribution of wild populations according to mean annual temperature and mean annual precipitation. The frequency distribution of the variables is depicted on the scatterplot axes. Coloured polygons depict the Whittaker’s biomes [39]. (B) Ecosystem productivity of wild populations based on NDVI and aridity index. (C) Principal component analysis showing the edaphoclimatic space of ancestral agriculture. The first two principal components (PC1 and PC2) explained 49% of the variance. The arrows indicate the loadings of the edaphoclimatic variables, and the ellipses are a multivariate t-distribution coloured according to clustered ecoregions. (D) Boxplot of the loadings of wild populations within the edaphoclimatic space. Abbreviations of variables: (MAP: Mean annual precipitation, MAT: Mean annual temperature, AI: Aridity index, OC: Organic carbon, CE: Electrical conductivity, NDVI: Normalized difference vegetation index). Asterisks denote median significantly different from zero (Wilcoxon signed-rank test, *P*-value <.05). Letters indicate significant differences amongst wild progenitors in each principal component (*P*-value adjusted with Bonferroni correction to account for multi-group comparisons, *P*-value <.05).

Our study provides a biogeographical characterization of the evolutionary origins of domesticated crops based on 125 populations of 10 CWPs (Fig. 1 and Table S2), distributed across all Whittaker biomes except for cold and high-precipitation temperate systems (Fig. 2A and B). The ecological niche of CWPs distributes along two dimensions that jointly describe variations in climate and soil, which delineate four markedly distinct ecoregions (Fig. 2C, Figs S3 and S4). The oligotrophic region of coastal xeric shrublands [encompassing wild cotton populations from the Yucatán Peninsula, Mexico [54]] separates from the remaining more fertile ecoregions, since it is characterized by the warmest temperatures, the lowest micronutrient concentrations, and the sandiest and saltiest soils (Tables S3 and S4). In these stressful environments, plant species most likely require physiological configurations with lower specific leaf area and higher tissue densities [55, 56]. On the opposite dimension, the ordination shows a gradient related to water and energy limitation, distinguishing amongst desert, temperate and tropical ecoregions (Fig. 2C and D). The desert ecoregion included arid and semi-arid grasslands with mild temperatures and alkaline soils, where the low rainfall constraint primary productivity and contributes to higher accumulation of total soil micronutrients [encompassed the wild barley and wheat from the Fertile Crescent (Israel and Iran [57]), and some populations of sunflower from North America (USA [58])]. The tropical seasonal forest and savanna ecoregion included warm and high-moisture environments, such as forests and wetlands, with mature acidic soils due to the intense weathering, and accumulation of SOC and inorganic nitrogen [included wild progenitors of little millet from the Indomalaya region (India [59]) and rice from the Yangtze and Yellow River valleys (China [60])]. The temperate dry forests and shrubland ecoregion was climatically intermediate, and presented higher soil concentrations of total phosphorus, but overall low fertility [agglutinated populations of maize and beans from Mesoamerica (Mexico [61, 62]), potato from the Central Andes (Bolivia [63]), and soya from China [64]]. The favourable climate conditions for plant growth in the tropical ecoregion are likely to yield larger size traits in CWPs and faster nutrient and carbon processing compared to progenitors in ecoregions with water or energy limitations such as deserts [55]. Whilst other factors such as environmental heterogeneity, phenotypic plasticity, intraspecific variability, or growth habits, may also influence changes in CWPs ecological performance [18, 55, 56], our biogeographical assessment of the evolutionary origins of major crops suggests different plant–soil functionalities amongst ecoregions. These ecological insights are useful to inform crop breeding and should be considered in research aimed at improving crops productivity and performance [18, 19].

### The soil core community of crop wild progenitors

**Figure 3:**
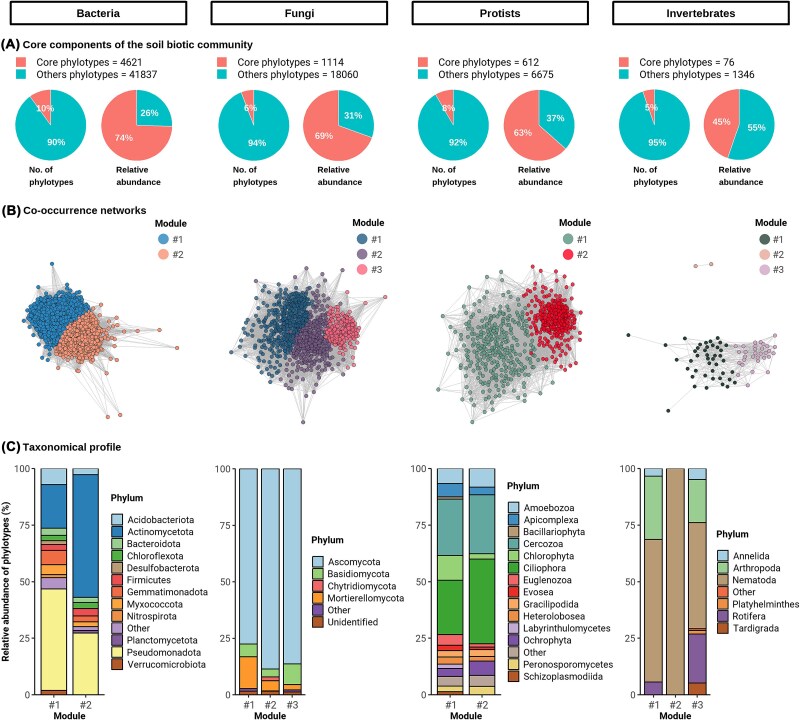
Core members of the soil biotic communities of ancestral agriculture (10% most abundant phylotypes present in soil samples from at least four crop wild progenitors). Plots in columns show data for bacteria, fungi, protists, and invertebrates. (A) Pie charts show the proportion of core phylotypes and their relative abundance compared to the remaining phylotypes. (B) Co-occurrence networks show significant relationships (edges with significance level *P* < .05) between core phylotypes (nodes), which are coloured by main modules (highly connected groups of nodes). (C) Barplots show the relative abundance of phylotypes in each module, based on the number of reads of core phylotypes at the phylum level. Relative abundances <1% were classified as “Other”.

We identified the core phylotypes (i.e., zOTUs) for the four kingdoms comprising the soil biotic community and assessed their organization into modules in co-occurrence networks (Fig. 3B and Table S1). We conducted the analyses on abundant and prevalent taxa within the bulk soil community because these are preferentially filtered by the CWPs in the rhizosphere and contributed the most to increased soil multifunctionality compared to less abundant phylotypes [44]. These core phylotypes accounted for a small portion of the total richness (5–10% of the total number of phylotypes), although dominated in abundance (45%–74% of the total number of reads, Fig. 3A). Moreover, a majority of bacterial core phylotypes were consistently found in soils from all CWPs, suggesting they possess generalist functions that support their survival across diverse environments (Fig. S5). Soil core phylotypes of fungi, protists, and invertebrates were associated with smaller sets of CWPs, which may indicate a greater differentiation and dependence on local conditions (Figs S6–S8). Although the centres of origin of CWPs may represent a biogeographically biased sample of soils worldwide, our results regarding core phylotypes aligns well with findings from global surveys (Fig. 3C), where Pseudomonadota, Actinomycetota, Ascomycota, Cercozoa, Ciliophora, and Nematoda are dominant phyla [11, 65–68]. Moreover, the soil core community included dominant genera of ecological and agricultural relevance, such as bacteria involved in phytohormones regulation (e.g. *Sphingomonas and Arthrobacter*), plant pathogenic fungi and oomycetes (e.g. *Alternaria, Fusarium, Pythium*), or plant feeding and parasitic nematodes (e.g. *Tylenchida*) (Figs S9–S12) [17, 35]. Our results indicate that these taxa, which likely compromise the health and productivity of CWPs, were unevenly distributed across the crop’s centres of origin.

**Figure 4:**
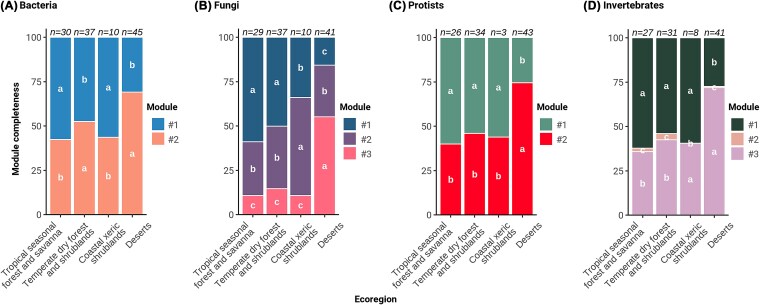
Module completeness of soil core members across the edaphoclimatic ecoregions. Colours indicate the averaged proportion of nodes belonging to a given module (*i.e.*, module completeness) of (A) bacterial, (B) fungal, (C) protist, and (D) invertebrate co-occurrence networks. *n* = number of wild populations surveyed. Letters indicate significant differences amongst modules within each ecoregion and kingdom (*α* = 0.05).

We identified the biogeographical patterns in soil biotic community by examining the proportion of nodes belonging to a given co-occurrence module across the edaphoclimatic ecoregions (i.e., module completeness, Fig. 4). Our results showed the existence of core phylotypes associated with deserts, since accounted for 55%–72% of total phylotypes in the ecoregion, and a second major module in tropical areas accounting for 34%–62%. These patterns may reflect interactions between soil taxa and plants, or the convergence of environmental preferences aligned with the edaphoclimatic gradient characterized by water and energy limitation [68, 69] (Fig. 2). In the desert ecoregion, the soil core community presented higher proportions of saprophytic fungi (Fig. S13), predatory and decomposer soil protists such as Amoebozoa and Ciliophora, as well as soil invertebrates acting as detritivores and predators like Annelida, Rotifera, and Tardigrada (Figs 3C and 4). Interestingly, the wild progenitors of wheat and barley stand out for being associated with specific core phylotypes and shaping a distinct soil community from that of the other CWPs, suggesting a higher degree of adaptation to the arid sites (Figs S5–S8 and S14). In these environments, the scarce availability of key soil resources such as organic carbon and nitrogen might convert CWP rhizosphere into fertility islands, where plant-derived organic inputs concentrate, leading to increased colonization by saprophytic life forms compared to bare soil areas [70]. Knowing the patterns of the soil core community in native regions is essential, as it has been shown to enhance both plant growth and health, as well as the effectiveness of synthetic communities [71, 72]. Phylotypes in the desert ecoregion where CWPs thrive (Figs 3B and C and 4), may help to address one of the most important challenges in dryland agriculture, namely climate change and land degradation [73]. Further research should explore the functional properties of these soil core phylotypes and how they influence plant evolutionary responses that ameliorate plant drought stress and enhance nutrient availability in the rhizosphere, thereby promoting plant growth [24]. In contrast, the soil core community in tropical and temperate ecoregions exhibited greater abundances of Acidobacteriota, fungal parasites, invertebrate parasitic protists like Labyrinthulomycetes, Apicomplexa, and kinetoplastids from the Euglenozoa phylum, as well as invertebrates such as Nematodes and Arthropoda (Figs 3C and 4 and Figs S9–S13). Our results showing increased abundances of parasites in soil communities from tropical compared to dryland ecoregions, can be attributed to several factors such as the higher temperature and moisture levels, a wider variety of host species, and the longer transmission seasons (Table S3) [68]. The wild progenitors of little millet and rice harbour specific core phylotypes that are predictably characteristic of this humid environment, with more anoxic and reducing conditions (e.g. optimal for Desulfobacterota). Wild cotton, originating from marine environments, also shapes a singular core community of soil protists (Fig. 1 and Figs S9–S14). In contrast, the wild progenitors of bean and soya, which are found in regions with intermediate conditions, function as a hub for the soil biotic communities, sharing core phylotypes with all CWPs (Figs S5–S8, S13, and S14). This evaluation of the soil core community of CWPs suggests differences in potential agricultural applications. The ecological module from the desert ecoregions is likely important for stimulating nutrient mineralization and enhancing plant nutrient availability, whereas those from tropical ecoregions may help control the pathogenic organisms contributing to plant health.

### Edaphoclimatic factors shaping modules of soil core biodiversity members

**Figure 5:**
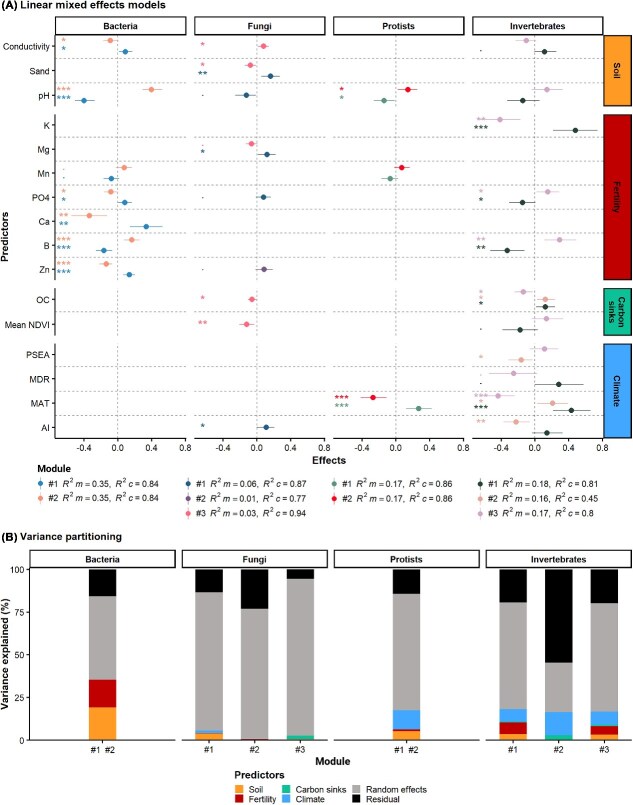
Influence of the environment on module assemblies of soil biotic communities of ancestral agriculture. (A) Environmental predictors for each module and microbial group (i.e., bacteria, fungi, protists, and invertebrates). Coloured point ranges indicate the main fixed effects (±95% confidence intervals). Significance levels: (^***^) *p* < 0.001, (^**^) *p* < 0.01, (^*^) *p* < 0.05, (.) *p* < 0.1. R^2^m and R^2^c—marginal and conditional *r*-squared in linear mixed models, respectively. (B) Variance partitioning by groups of environmental predictors. Total variance (*R*^2^) partitioned into residual variance, random effects variance (crop wild progenitor identity), and the fraction attributable to unique and shared variance by each group of potential predictors (soil properties: pH, conductivity, sand; soil fertility: NH_4_^+^, NO_3_^−^, PO_4_^−^, Ca, B, K, Mg, Zn, Fe, Mn, Mo; potential carbon sinks: Mean NDVI—normalized difference vegetation index, SOC—soil organic carbon; climate: MAT—mean annual temperature, MDR—mean diurnal range temperature, AI—aridity index). A single stacked bar of explained variance is shown when there are only two microbial modules in the co-occurrence network because their relative abundances are complementary.

We investigated the factors that determine variations in the abundances of the ecological modules within the soil core community of CWPs (Fig. 5). Soil pH was the main driver of the bacterial modules, along with soil salinity and several micronutrients. This suggests that certain bacterial taxa abundant in the tropical and coastal shrubland ecoregions are selected for tolerance to acidic soils, as is the case of Acidobacteriota, and Pseudomonadota of the Xanthomonadaceae and Burkholderiaceae families, and salinity as Gammaproteobacteria of the family Alteromonadaceae and Bacteroidota of the families Flavobacteriaceae and Rhodothermaceae [67, 74]. Fungal modules were mainly influenced by sand content, zinc concentration, primary productivity, and aridity. Our results confirm that soil-dwelling fungi are significantly affected by vegetation and climate factors [65, 66]. These different environmental preferences amongst fungal modules may be related to variations in fungal functional groups [65]. As we said, there was less diversity of fungal parasites, but higher of saprophytic fungi, in areas with higher aridity and lower productivity (Fig. S13). Soil protists are distributed as a function of mean annual temperature and, to a lesser extent, soil pH. This indicates differences in the dominant mode of energy acquisition by soil protist communities [68], with increasing temperatures and lower pH favouring greater proportions of parasites as is the case of Labyrinthulomycetes, Apicomplexa, and kinetoplastids, in detriment of other free living heterotrophs such as Amoebozoa and Cercozoa. Soil invertebrates were influenced by a higher number of environmental predictors, likely due to complex interactions amongst trophic levels [69]. Our results are consistent with those of Bastida *et al*. [11] indicating climate, including its seasonal and daily variability, and vegetation productivity as main drivers of soil invertebrate communities. Taken together, we found that the sets of environmental factors shaping the biological soil community of CWPs varied amongst kingdoms, highlighting the complexity of community-level assembly in native soil environments, and pointing to pH as a common predictor to all of them. Our results are meaningful to guide soil microbial inoculation since we decipher the environmental conditions that can promote particular modules of the ancestral core microbiome [16]. This knowledge will allow the use of specific microbial inoculants adapted to local conditions ensuring their environmental persistence [16, 17]. The current political and economic context seems adequate to harness the potential of these soil core community of ancestral agriculture, as the industry of agricultural microbial products is increasing at a global annual rate of 17% [3].

Interestingly, the identity of CWPs was the major source of variation amongst ecological modules of the soil core community (i.e., random effects in the models in Fig. 5). This result may suggest that interactions between plant hosts and the soil microbiome shape module abundances at the local scale [75] (Fig. S14), where the active selection of distinct soil microbiomes takes place [76]. These selection processes could potentially be triggered by the plant phenotypes, or these host phenotypes could be the consequence of plant-independent ecological interactions within the native soil community that do not occur in agricultural systems [8]. Hence, distinct wild populations within ecoregions are potential reservoirs of the co-evolutionary relationships between CWPs and soil organisms [2, 8].

## Conclusions

We found that the populations of CWPs were distributed across widely diverse edaphoclimatic conditions that clustered into four ecoregions. The coastal xeric shrubland ecoregion showed saline sandy soils with low fertility, whilst desert, tropical seasonal forest, and savanna ecoregions featured more fertile soils, although they differed significantly in aridity, soil pH, carbon storage potential, and nutrients concentrations. The ecological modules of the soil core community exhibited distinct taxonomical profiles and abundance patterns across ecoregions, indicating shifts in the life-history strategies and functional traits of soil members. For instance, our analyses indicated that soils from tropical regions harboured greater proportions of acidophilic bacteria, as well as fungal and protist parasites, whereas deserts harboured higher abundances of saprophytic fungi and heterotrophic protists. Our results also suggest that CWPs host unique and diverse soil microbiomes, highlighting the wild populations as fundamental conservation units for the preservation of eco-evolutionary relationships between CWPs and their associated soil biota. This multi-kingdom characterization of soil biodiversity at the geographical regions where agriculture originated can serve as a foundation for targeted microbiome rewilding, and can further inform integrated soil conservation strategies, from plant genetic and soil diversities to their co-evolutionary interactions.

## Supplementary Material

SI_R3_ycaf143

## Data Availability

*Extended data* Amplicon sequencing data generated in this study is available through the European Nucleotide Archive (ENA) under the project accession number PRJEB89460. The specific run accession numbers are: ERR14986789-ERR14986913 for 16S rRNA amplicons, ERR14987213-ERR14987337 for ITS region amplicons, and ERR14987347-ERR14987471 for 18S rRNA gene amplicons. The data and scripts used to generate the analyses and figures presented in this study are openly available on Figshare: https://doi.org/10.6084/m9.figshare.26990866.v1 [77].
