# Correction to: Native edaphoclimatic regions shape soil communities of crop wild progenitors

**DOI:** 10.1093/ismeco/ycag088

**Published:** 2026-06-17

This is a correction to: María José Fernández-Alonso, Miguel de Celis, Ignacio Belda, Javier Palomino, Carlos García, Juan Gaitán, Jun-Tao Wang, Luis Abdala-Roberts, Fernando D Alfaro, Diego F Angulo-Pérez, Manoj-Kumar Arthikala, Danteswari Chalasani, Jason Corwin, Duan Gui-Lan, Antonio Hernandez-Lopez, Kalpana Nanjareddy, Siddaiah Chandra Nayaka, Babak Pasari, Thanuku Samuel Sampath Kumar Patro, Appa Rao Podile, Teresa Quijano-Medina, Daniela S Rivera, Pullabhotla Venkata Subba Rama Narshima Sarma, Salar Shaaf, Pankaj Trivedi, Qingwen Yang, Yue Yin, Eli Zaady, Yong-Guan Zhu, Brajesh K Singh, Manuel Delgado-Baquerizo, Pablo García-Palacios, Ruben Milla, Native edaphoclimatic regions shape soil communities of crop wild progenitors, *ISME Communications*, Volume 5, Issue 1, January 2025, ycaf143, https://doi.org/10.1093/ismeco/ycaf143

The following changes have been made to the originally published paper. Markup and statements on the equal contribution of the first two authors to this paper have been added.

