# Supplementary material for: Native edaphoclimatic regions shape soil communities of crop wild progenitors
Source: ISME Commun. 2025 Sep 24;5(1):ycaf143. doi: 10.1093/ismeco/ycaf143 (PMC12515042; doi:10.1093/ismeco/ycaf143)
Supplement: SI_R3_ycaf143 [file si_r3_ycaf143.pdf]

Native edaphoclimatic regions shape soil communities of crop wild progenitors

Supplementary information

María José Fernández-Alonso[1,2a*], Miguel de Celis[3*], Ignacio Belda[4], Javier Palomino[1], Carlos García[5], Juan Gaitán[6], Jun-Tao Wang[7,8,9], Luis Abdala-Roberts[10], Fernando D Alfaro[11], Diego F Angulo-Pérez[12], Manoj-Kumar Arthikala[13], Danteswari Chalasani[14], Jason Corwin[15], Gui-Lan Duan[16], Antonio Hernandez-Lopez[13], Kalpana Nanjareddy[13], Siddaiah Chandra Nayaka[17], Babak Pasari[18], Thanuku Samuel Sampath Kumar Patro[19], Appa Rao Podile[14], Teresa Quijano-Medina[10], Daniela S Rivera[11], Pullabhotla Venkata Subba Rama Narshima Sarma[14], Salar Shaaf[20], Pankaj Trivedi[15], Qingwen Yang[21], Yue Yin[16,22], Eli Zaady[23], Yong-Guan Zhu[16], Brajesh K Singh[7,8], Manuel Delgado-Baquerizo[22a], Pablo García-Palacios[3,24a], Ruben Milla[1,25a]

[1] Area of Biodiversity and Conservation, Department of Biology and Geology, Physics and Inorganic Chemistry, Rey Juan Carlos University, C/ Tulipán s/n, 28933 Móstoles, Spain
[2] Departamento de Geología y Geoquímica, Facultad de Ciencias, Universidad Autónoma de Madrid, 28049, Madrid, Spain
[3] Departamento de Suelo, Planta y Calidad Ambiental, Instituto de Ciencias Agrarias, Consejo Superior de Investigaciones Científicas, 28006 Madrid, Spain
[4] Department of Genetics, Physiology and Microbiology, Faculty of Biology, Complutense University of Madrid, 28040 Madrid, Spain
[5] Department of Soil and Water Conservation and Organic Waste Management, CEBAS-CSIC, 30100 Murcia, Spain
[6] Instituto de Suelos - INTA Castelar, CONICET, Universidad Nacional de Luján, 6700 Luján, Buenos Aires, Argentina
[7] Hawkesbury Institute for the Environment, Western Sydney University, Penrith, NSW 2751, Australia
[8] Global Centre for Land-Based Innovation, Western Sydney University, Penrith, NSW 2751, Australia
[9] School of Science, Western Sydney University, Penrith, NSW 2751, Australia
[10] Departamento de Ecología Tropical, Campus de Ciencias Biológicas y Agropecuarias, Universidad Autónoma de Yucatán, 97000 Mérida, Yucatán, Mexico
[11] GEMA Center for Genomics, Ecology and Environment, Universidad Mayor, Huechuraba, Santiago, Chile
[12] Unidad de Recursos Naturales, Centro de Investigación Científica de Yucatán, 97205 Mérida, Yucatán, México
[13] Ciencias Agrogenómicas, Escuela Nacional de Estudios Superiores Unidad León, Universidad Nacional Autónoma de México, León 37689, Guanajuato, Mexico
[14] Department of Plant Sciences, School of Life Science, University of Hyderabad, Hyderabad, 500046, Telangana, India
[15] Microbiome Network and Department of Agricultural Biology, Colorado State University, Fort Collins, CO 80523-1177, USA
[16] State Key Laboratory of Urban and Regional Ecology, Research Center for Eco-Environmental Sciences, Chinese Academy of Sciences, Beijing 100085, China
[17] Department of Studies in Biotechnology, University of Mysore, Manasagangotri, Mysuru, Karnataka, India
[18] Department of Agronomy and Plant Breeding, Islamic Azad University, Sanandaj Branch, Sanandaj 6616935391, Iran
[19] Agricultural Research Station (ACRIP center-small millets), Acharya NG Ranga Agricultural University, Vizianagaram, 535001, Andhra Pradesh, India
[20] Leibniz Institute of Plant Genetics and Crop Plant Research (IPK), 06466 Seeland, Germany
[21] National Key Facility for Crop Gene Resources and Genetic Improvement, Institute of Crop Sciences, Chinese Academy of Agricultural Sciences, Beijing 100081, China
[22] Laboratorio de Biodiversidad y Funcionamiento Ecosistémico, Instituto de Recursos Naturales y Agrobiología de Sevilla (IRNAS), CSIC, 41012 Sevilla, Spain
[23] Katif Research & Development Center, Sdot Negev, Netivot, 8771002, Israel
[24] Department of Plant and Microbial Biology, University of Zurich, CH-8008 Zurich, Switzerland
[25] Global Change Research Institute, Rey Juan Carlos University, 28933 Móstoles, Spain
[*] These authors contributed equally to this work

[a] Corresponding authors

María José Fernández Alonso, Departamento de Geología y Geoquímica, Universidad Autónoma de Madrid, Avd. Francisco Tomás y Valiente 7, Madrid, Spain, 28049, Spain. Email: mariajose.fernandez@uam.es, https://orcid.org/0000-0001-7874-6775

Manuel Delgado-Baquerizo, Instituto de Recursos Naturales y Agrobiología de Sevilla (IRNAS-CSIC), Av. Reina Mercedes 10, Sevilla 41012, Spain. Email: m.delgadobaquerizo@gmail.com, https://orcid.org/0000-0002-6499-576X;

Pablo García-Palacios, Instituto de Ciencias Agrarias, C. de Serrano 115b, 28006 Madrid, Spain. Email: pablo.garcia@ica.csic.es, https://orcid.org/0000-0002-6367-4761

Rubén Milla, Universidad Rey Juan Carlos, C. Tulipán s/n, Móstoles 28933, Spain. Email: ruben.milla@urjc.es, https://orcid.org/0000-0001-8912-4373

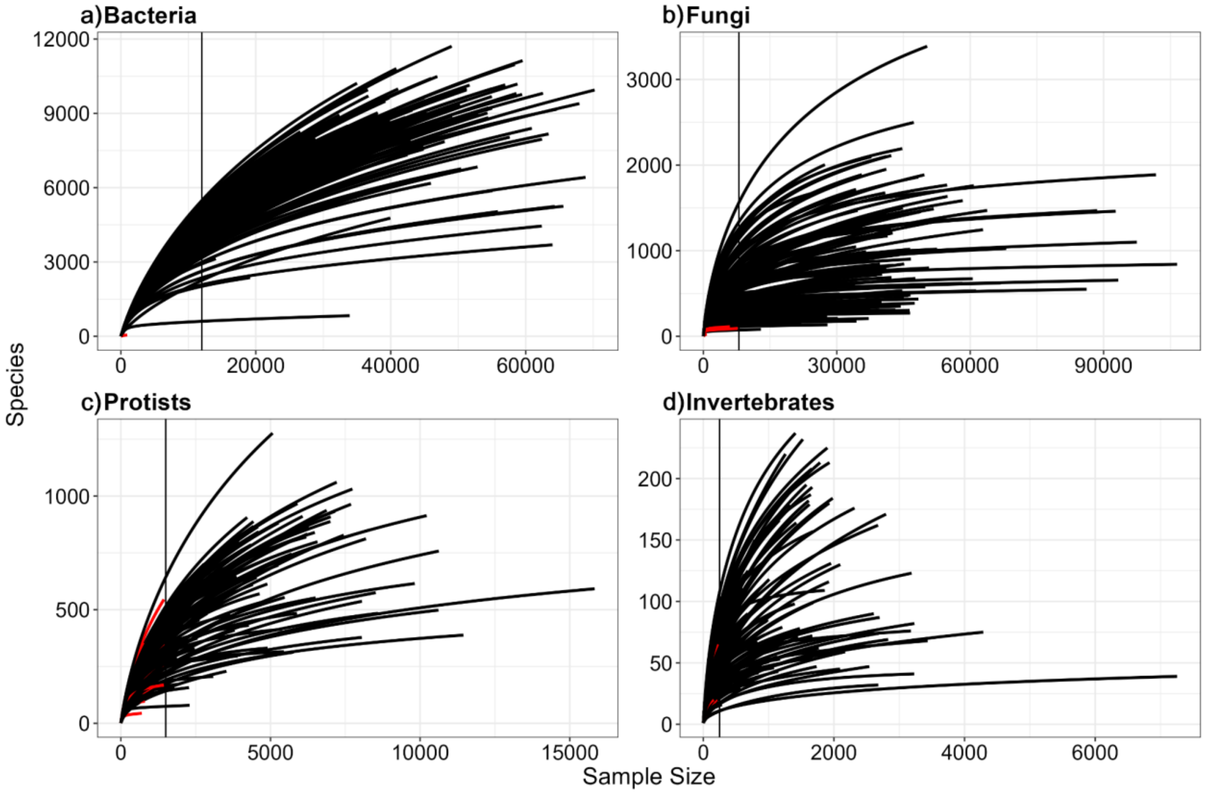

Figure S1 Rarefaction curves for each kingdom. Panels **a-d** show **rarefaction curves for bacteria, fungi, protists, and invertebrates, respectively.** In each panel, the *y*-axis represents the number of unique zOTUs, while the *x*-axis indicates the cumulative number of sequences per sample (black curves). The vertical line marks the rarefaction threshold applied for each group. Red curves highlight samples with low sequencing depth that were excluded from downstream analysis.

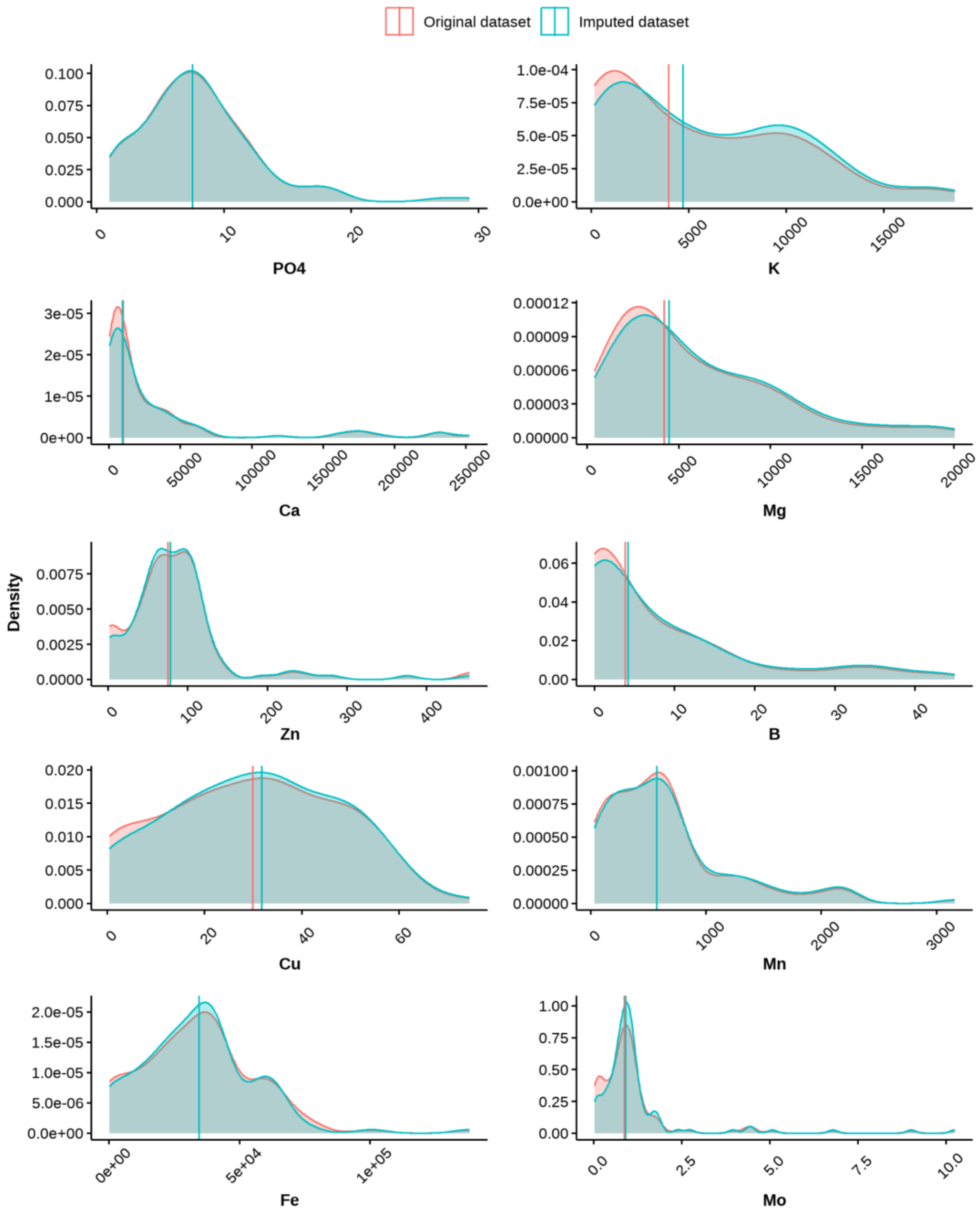

Figure S2 Density plot of soil variables. Colours indicate the original and the imputed datasets. Vertical lines represent the median values for each group, with data measured in mg kg^{-1}.

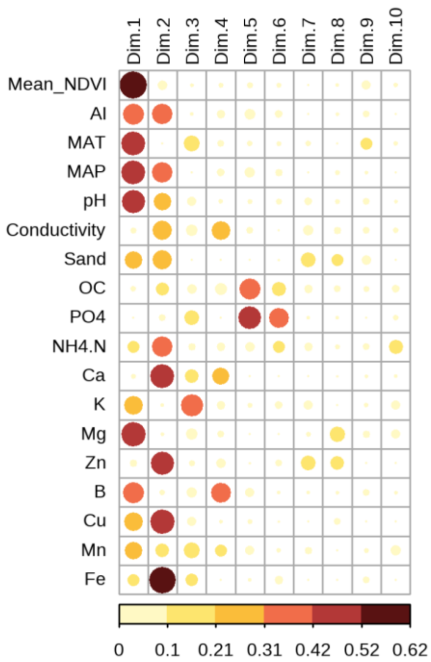

Figure S3 Quality representation of the Principal Components Analysis. Heatmap of the square cosine of the variables and dimensions (Figure 2c).

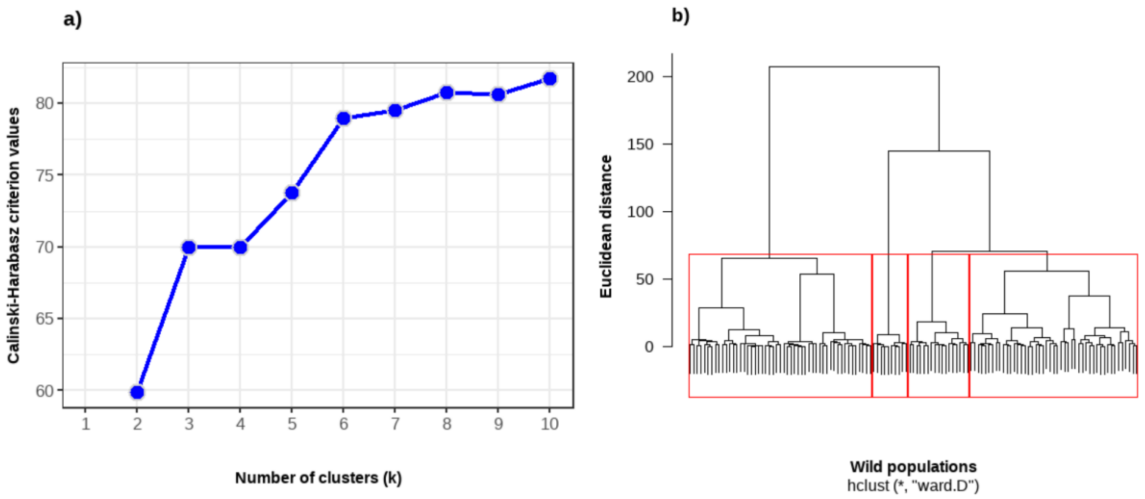

Figure S4 Quality evaluation of clustering algorithms. a) Calinski-Harabasz criterion values for different numbers of modules (k) using the K-means clustering algorithm. **b)** Cluster diagram of the ecoregions of ancestral agriculture based on the Euclidean distance between wild populations in the Principal Component Analysis (based on the first 5 Principal Components multiplied by their eigenvalues). The red box indicates the number of optimal modules selected (k = 4).

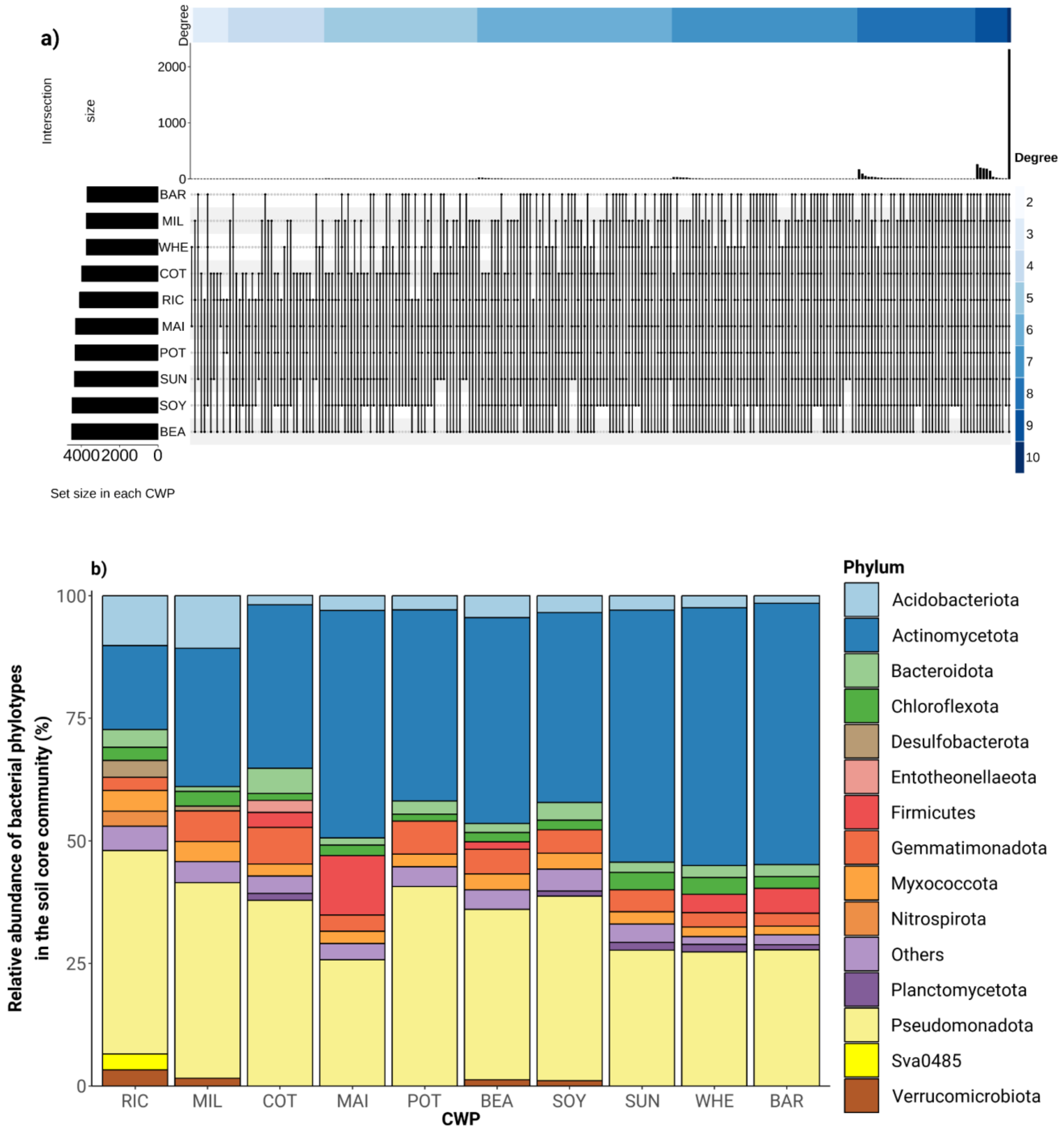

Figure S5 Distribution of bacterial phylotypes in the soil core community of crop wild progenitors (CWP). a) Upset plot depicting the shared bacterial phylotypes of the soil core community among CWP. The vertical barplot displays the intersection sizes, representing the number of bacterial phylotypes shared by combinations of CWP (Degree). In the centre, a matrix of black dots connected by vertical lines indicates which specific set of CWP shared the bacterial phylotypes. The horizontal barplot on the left displays the total number of core bacterial phylotypes detected in each CWP. **b)** Barplot shows the relative abundance of bacterial phylotypes in each CWP, based on the number of reads of core phylotypes at the phylum level. Relative abundances <1% were classified as 'Others'.

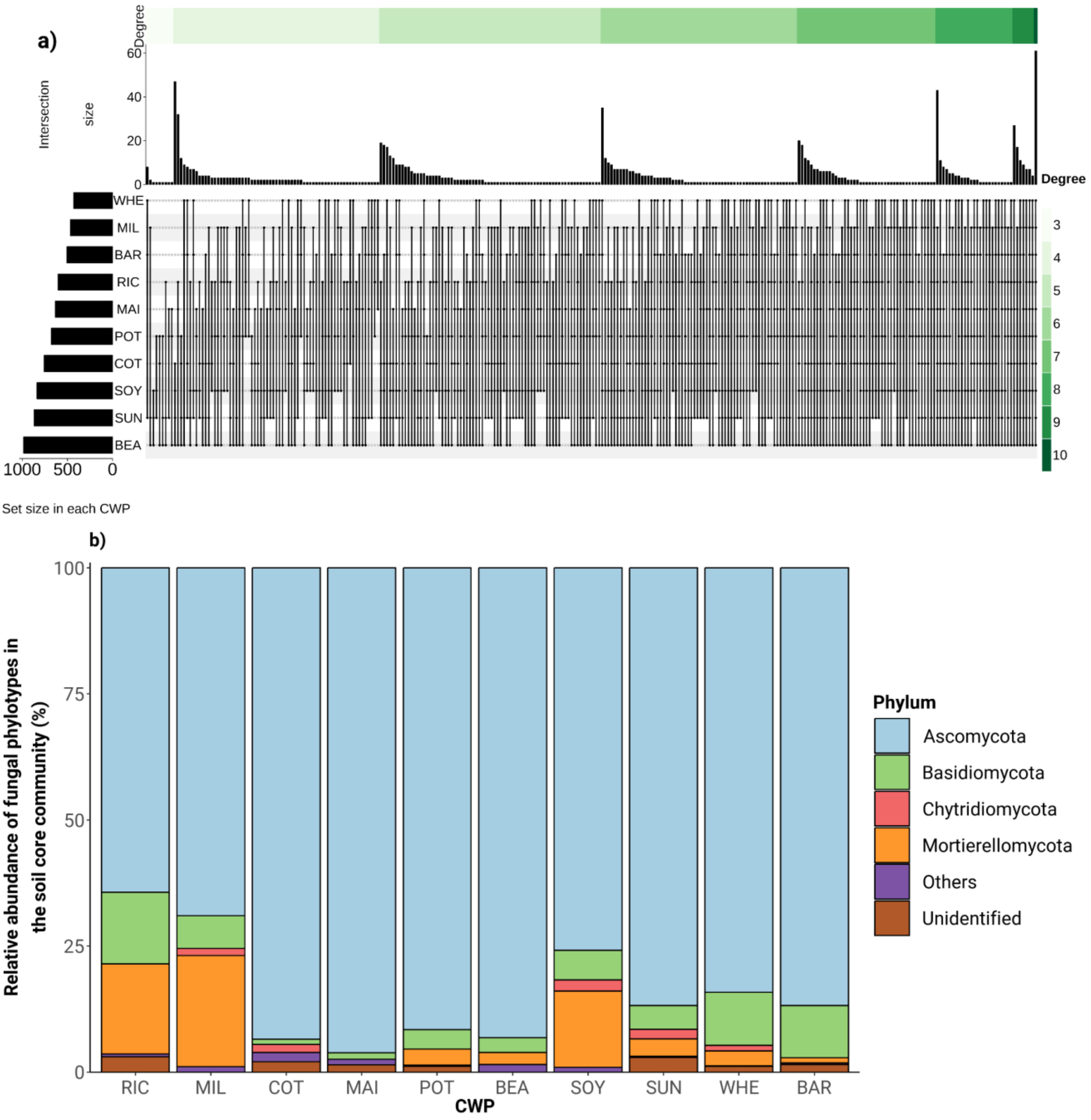

Figure S6 Distribution of fungal phylotypes in the soil core community of crop wild progenitors (CWP). a) Upset plot depicting the shared fungal phylotypes of the soil core community among CWP. The vertical barplot displays the intersection sizes, representing the number of fungal phylotypes shared by combinations of CWP (Degree). In the centre, a matrix of black dots connected by vertical lines indicates which specific set of CWP shared the fungal phylotypes. The horizontal barplot on the left displays the total number of core fungal phylotypes detected in each CWP. **b)** Barplot shows the relative abundance of fungal phylotypes in each CWP, based on the number of reads of core phylotypes at the phylum level. Relative abundances <1% were classified as ‘Others’.

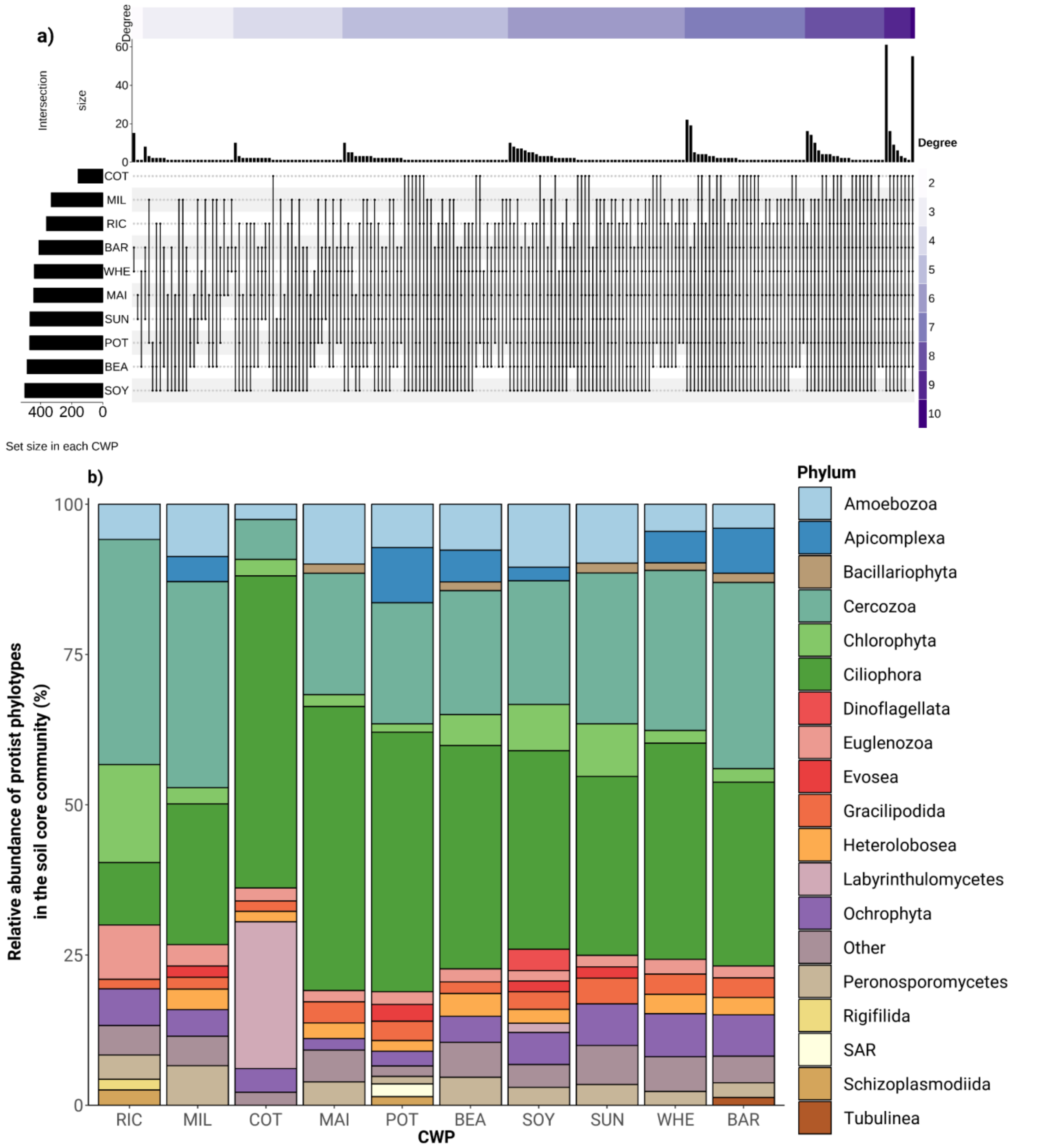

Figure S7 Distribution of protist phylotypes in the soil core community of crop wild progenitors (CWP). **a)** Upset plot depicting the shared protist phylotypes of the soil core community among CWP. The vertical barplot displays the intersection sizes, representing the number of protist phylotypes shared by combinations of CWP (Degree). In the centre, a matrix of black dots connected by vertical lines indicates which specific set of CWP shared the protist phylotypes. The horizontal barplot on the left displays the total number of core protist phylotypes detected in each CWP. **b)** Barplot shows the relative abundance of protist phylotypes in each CWP, based on the number of reads of core phylotypes at the phylum level. Relative abundances <1.2% were classified as 'Other'.

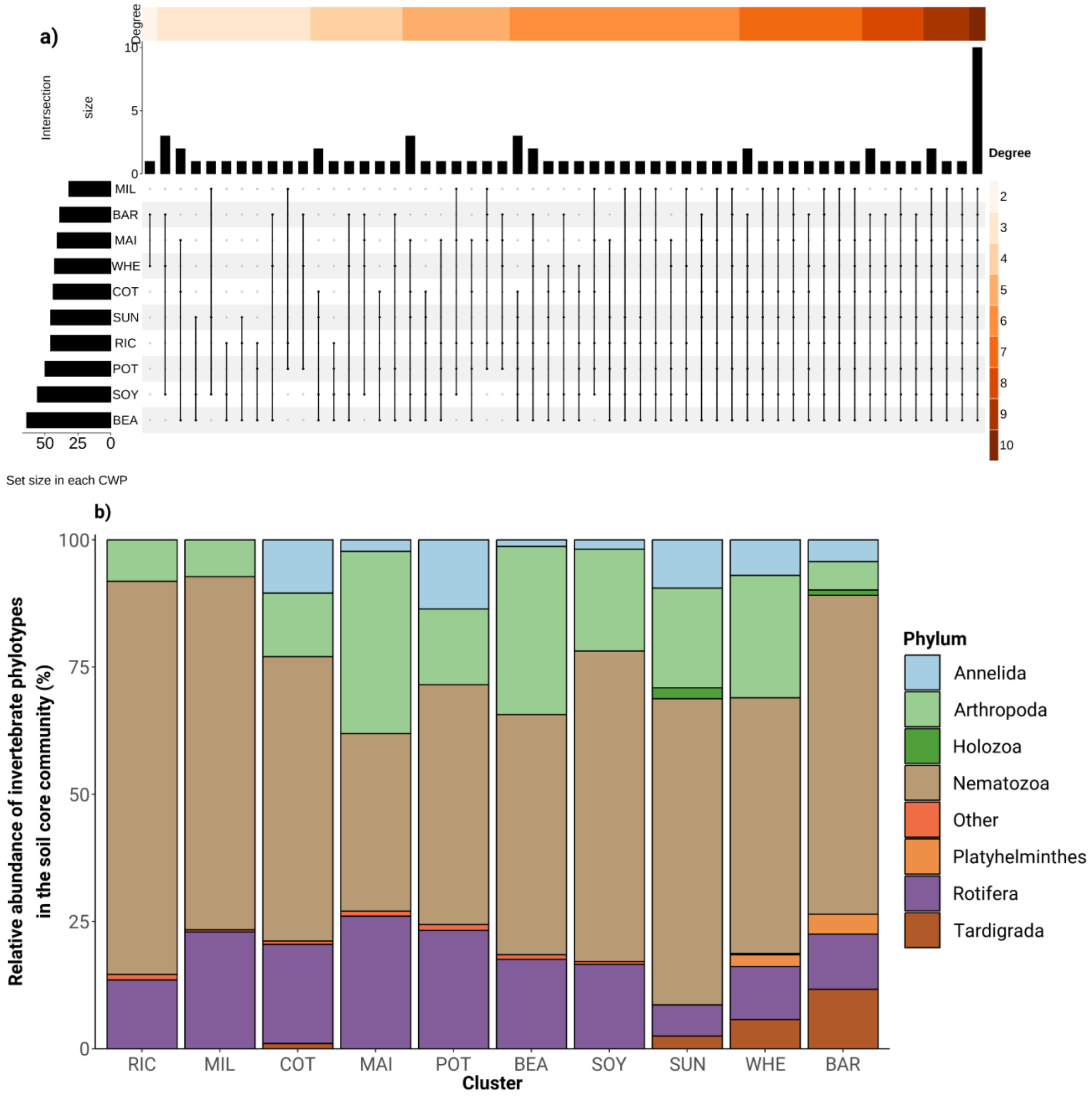

Figure S8 Distribution of invertebrate phylotypes in the soil core community of crop wild progenitors (CWP). a) Upset plot depicting the shared invertebrate phylotypes of the soil core community among CWP. The vertical barplot displays the intersection sizes, representing the number of invertebrate phylotypes shared by combinations of CWP (Degree). In the centre, a matrix of black dots connected by vertical lines indicates which specific set of CWP shared the invertebrate phylotypes. The horizontal barplot on the left displays the total number of core invertebrate phylotypes detected in each CWP. **b)** Barplot shows the relative abundance of invertebrate phylotypes in each CWP, based on the number of reads of core phylotypes at the phylum level. Relative abundances <1% were classified as 'Other'.

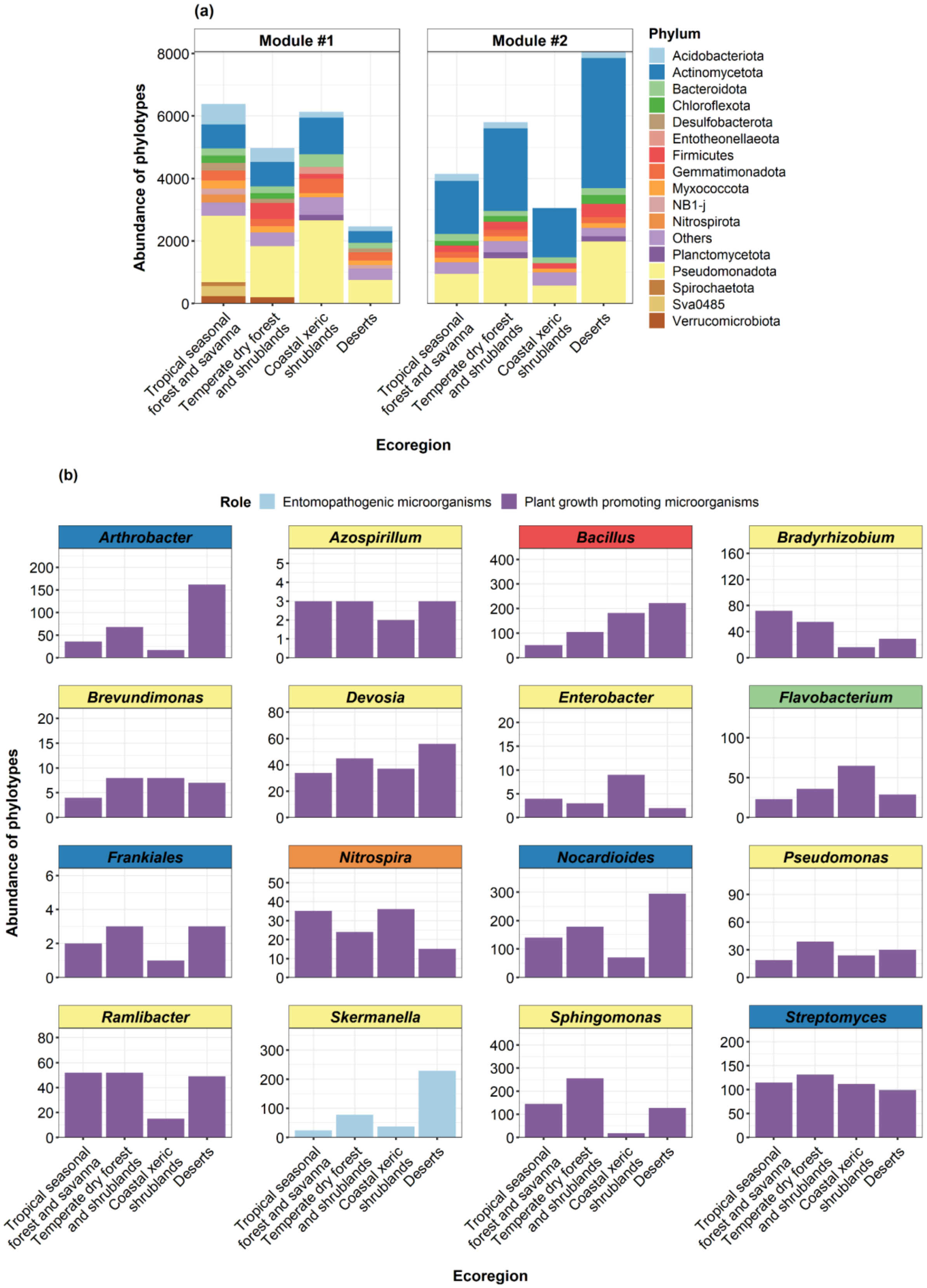

Figure S9 Soil core bacteria by ecoregion. Data represent the averaged number of reads. **(a)** Core phylotypes at the phylum level in each module of the bacterial co-occurrence networks (Others < 1% of reads). **(b)** Major bacterial genera are in abundance, along with other minor genera reported in the literature for their relevance in agriculture. The background colour of the title in each panel indicates the phylum (see legend in panel a).

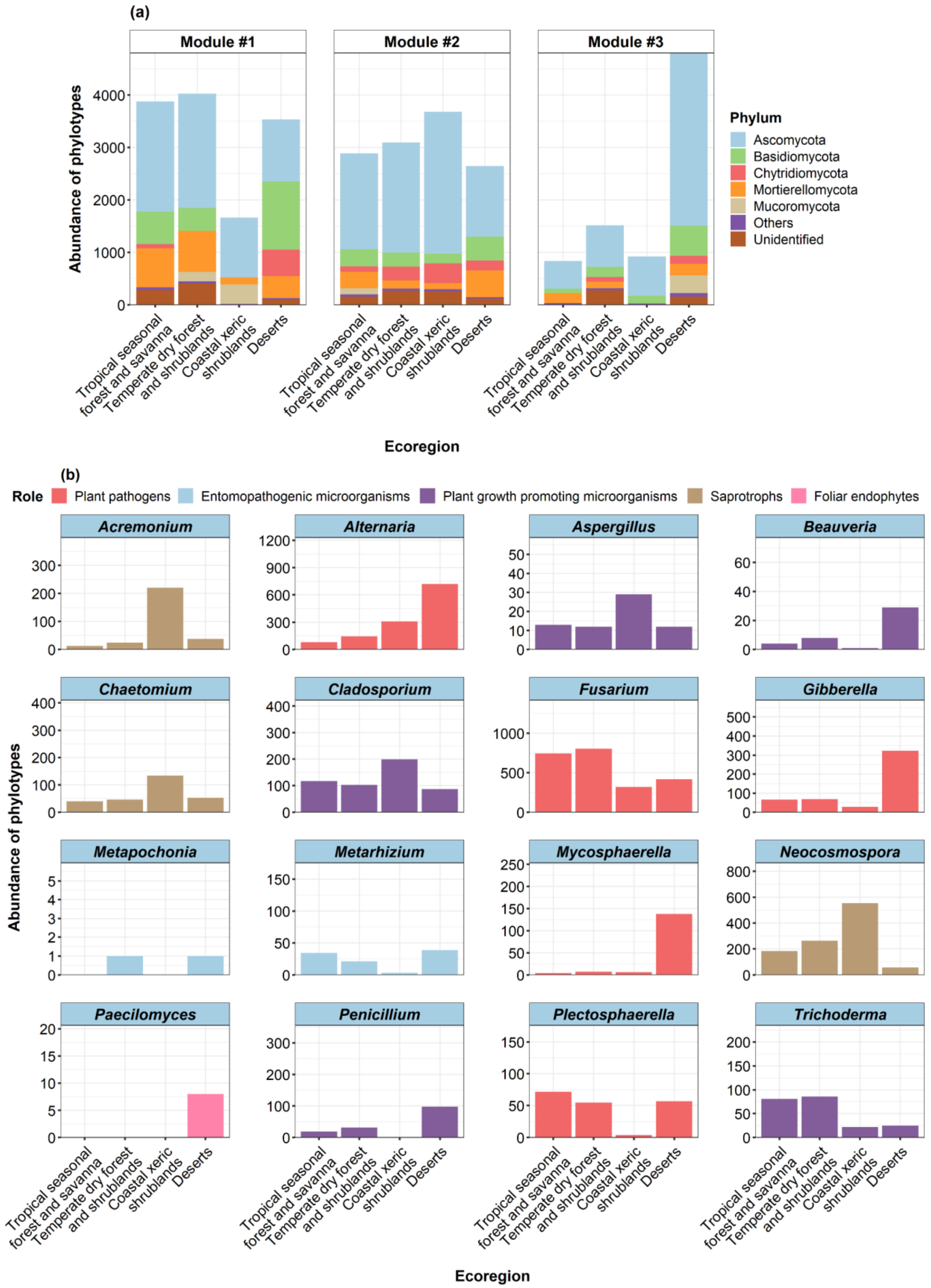

Figure S10 Soil core fungi by ecoregion. Data represent the averaged number of reads. **(a)** Core phylotypes at the phylum level in each module of the fungal co-occurrence networks (Others < 1% of reads). **(b)** Major fungal genera are in abundance, along with other minor genera reported in the literature for their relevance in agriculture. The background colour of the title in each panel indicates the phylum (see legend in panel a).

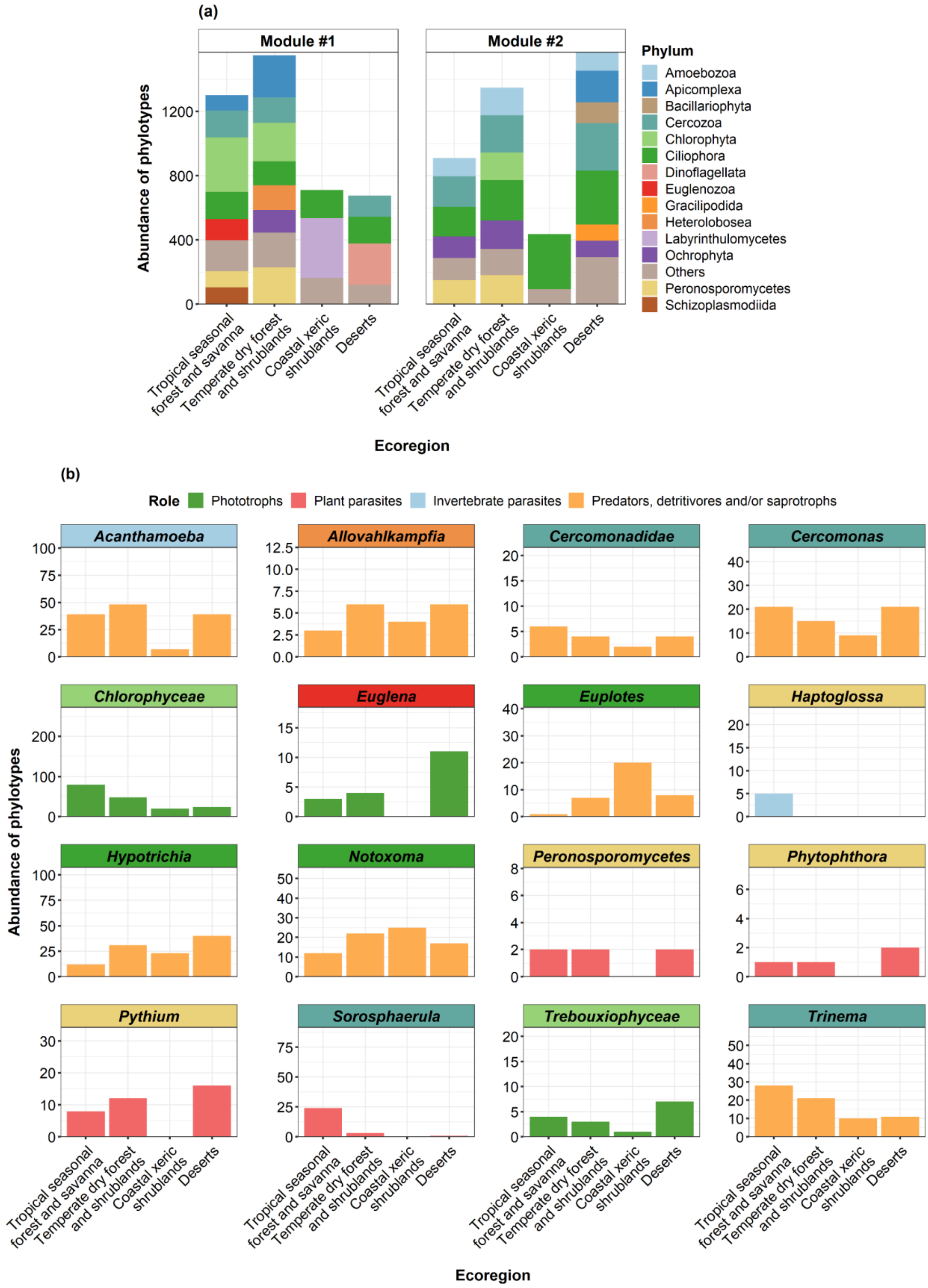

Figure S11 Soil core protists by ecoregion. Data represent the averaged number of reads. **(a)** Core phylotypes at the phylum level in each module of the protist co-occurrence networks (Others < 6% of reads). **(b)** Major protist taxa in abundance, along with other minor taxa reported in the literature for their relevance in agriculture. The background colour of the title in each panel indicates the phylum (see legend in panel a).

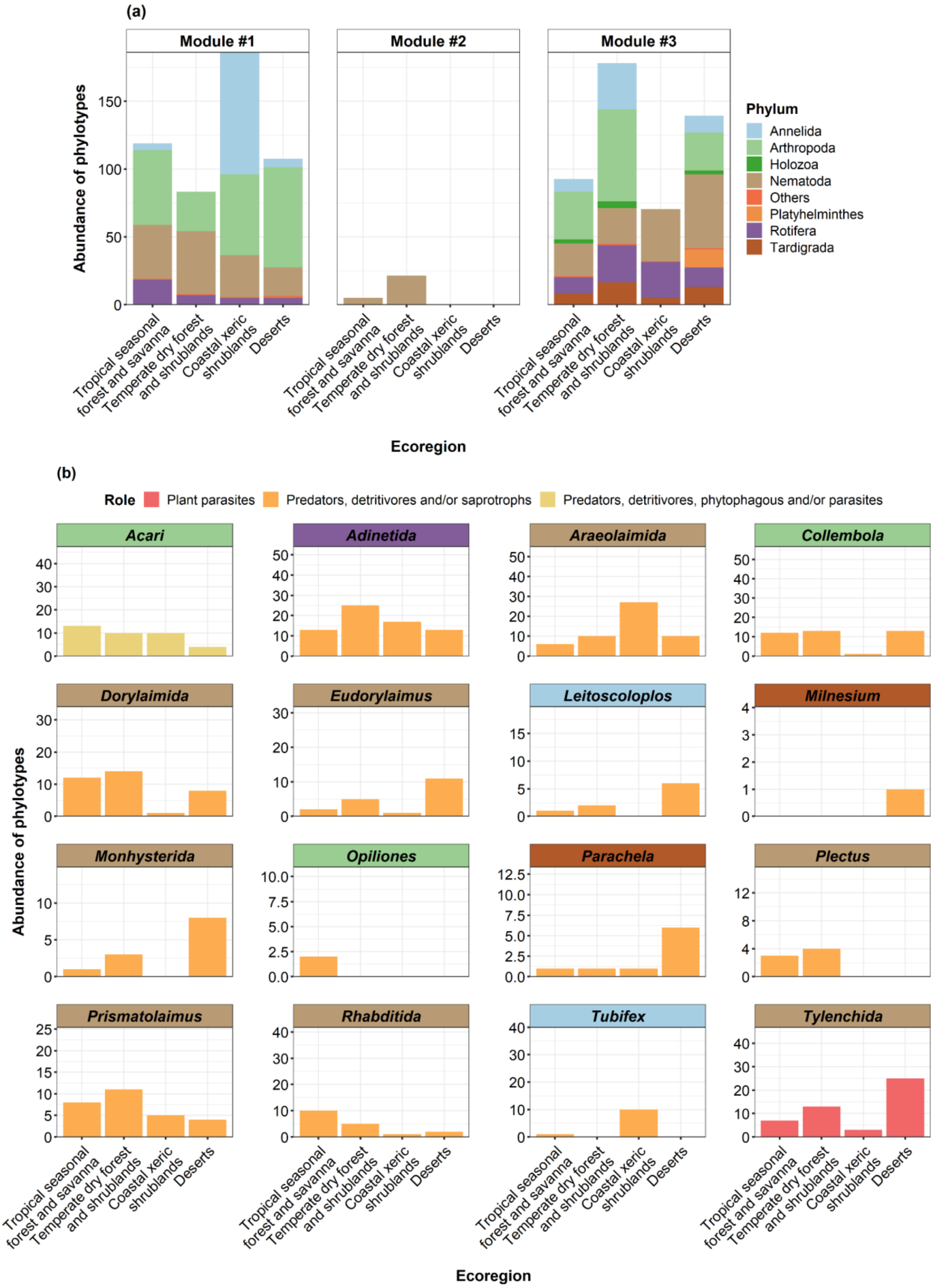

Figure S12 Soil core invertebrates by ecoregion. Data represent the averaged number of reads. **(a)** Core phylotypes at the phylum level in each module of the invertebrate co-occurrence networks (Others < 1% of reads). **(b)** Major invertebrate taxa in abundance, along with other minor taxa reported in the literature for their relevance in agriculture. The background colour of the title in each panel indicates the phylum (see legend in panel a).

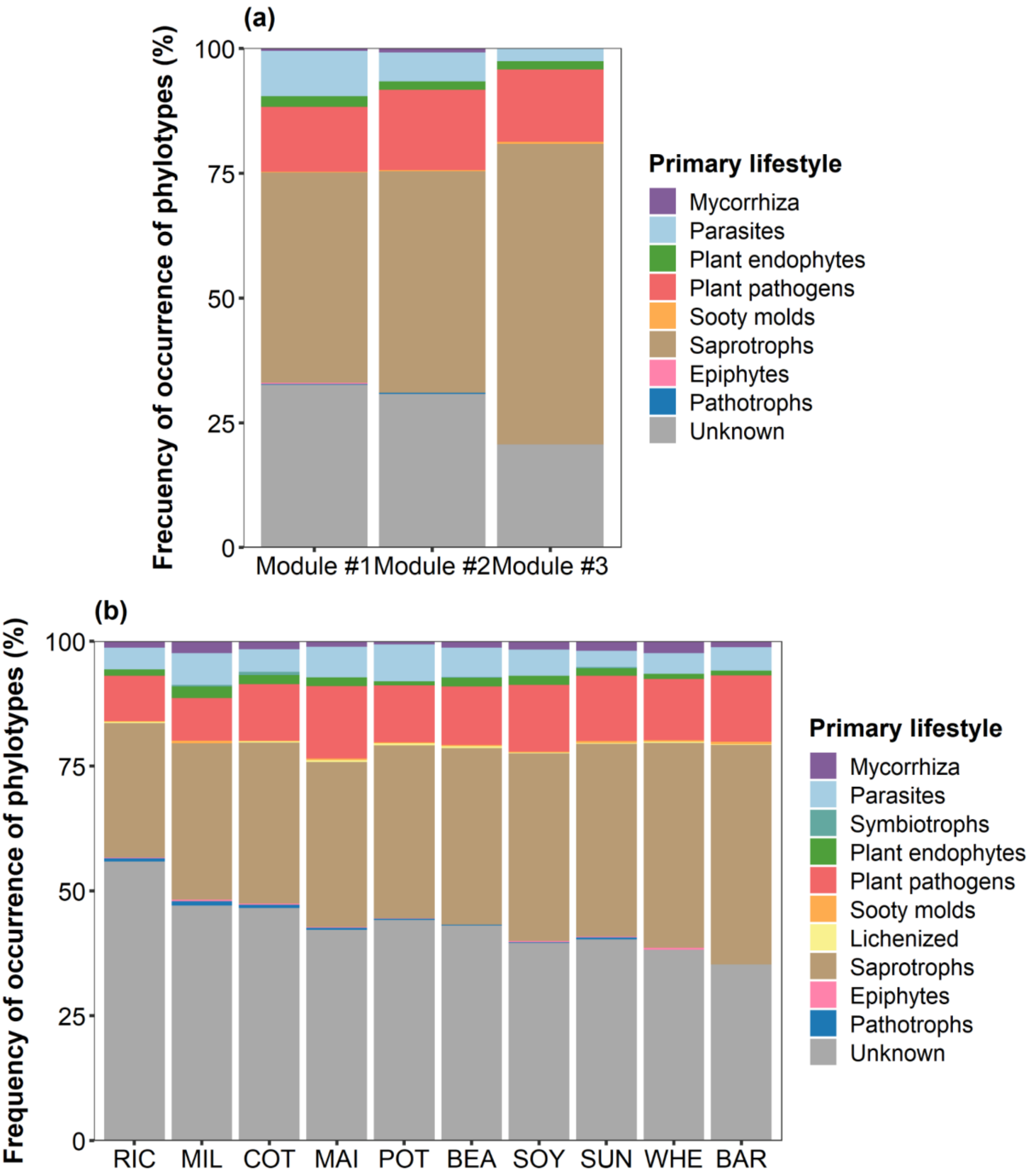

Figure S13 Potential lifestyle of the soil fungal community. a) Potential lifestyles of soil core fungi in modules of the co-occurrence networks. **b)** Potential lifestyles of whole soil fungi (core and non-core phylotypes) by crop wild progenitors (see abbreviations in Figure 1). Elaborated using the FungalTraits v1.2 database and the phylogenetic markers FITS7/ITS4 (Põlme et al., 2020).

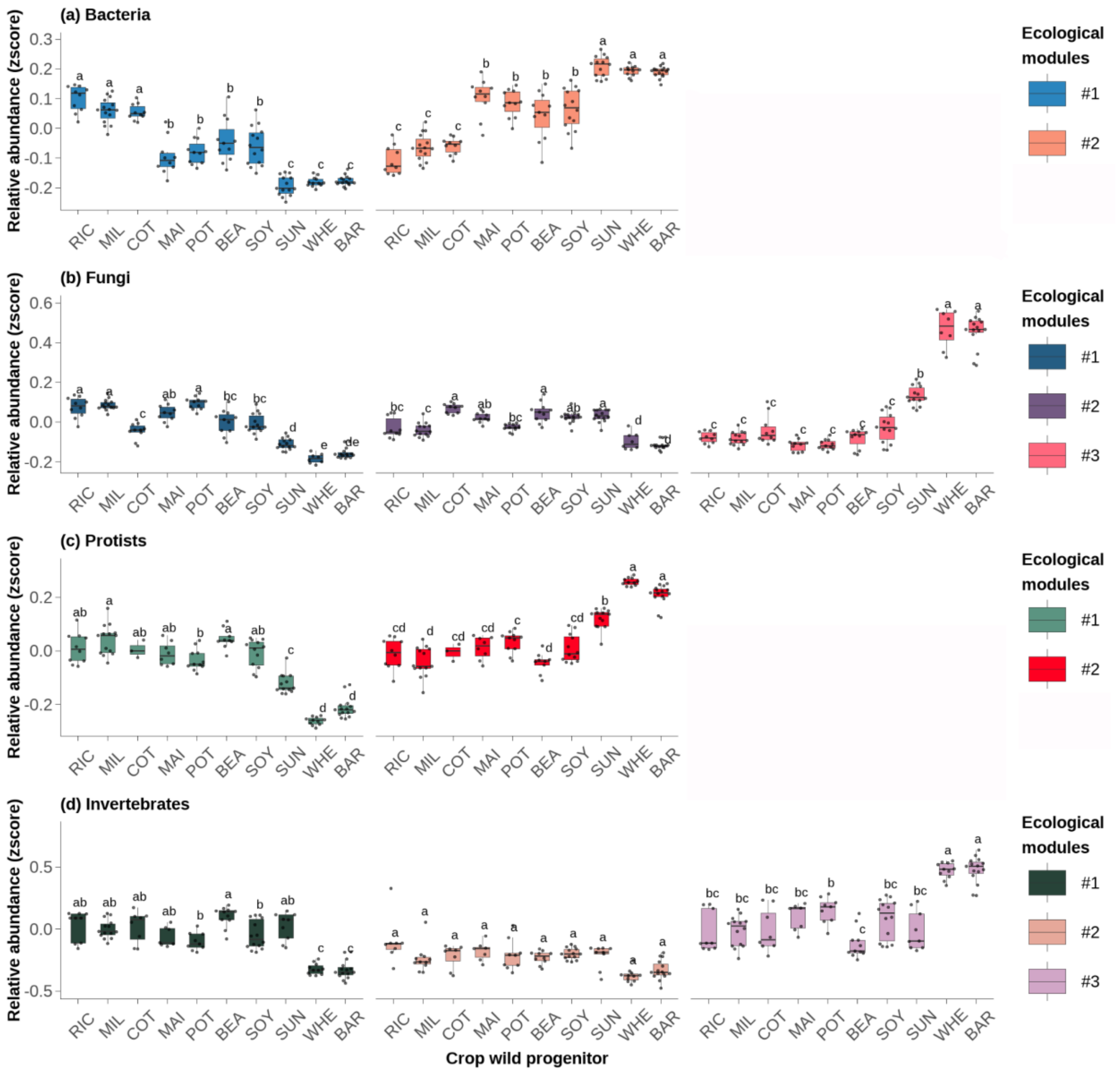

Figure S14 Relative abundances of ecological modules in the soil core microbiome across crop wild progenitors. a-d) The balanced contribution of core phylotypes is represented using the z-score (see abbreviations in Figure 1). Letters indicate significant differences among wild progenitors within each module (p-value adjusted with Bonferroni correction to account for multi-group comparisons, p-value < 0.01).

Table S1 Topological properties of microbial co-occurrence networks.

Microbial group	Nodes	Edges	Edge density	Modularity	Clustering coefficient	Modules	Num. phylotypes
Bacteria	4,611	1,561,032	0.15	0.34	0.43		
						#1	2387 (52%)
						#2	2224 (48%)
Fungi	1,114	168,860	0.27	0.27	0.58		
						#1	430 (39%)
						#2	428 (38%)
						#3	256 (23%)
Protists	612	36,268	0.19	0.29	0.68		
						#1	304 (50%)
						#2	308 (50%)
Invertebrates	76	705	0.25	0.34	0.62		
						#1	43 (57%)
						#2	2 (3%)
						#3	31 (41%)

Table S2 Coordinates of the 125 natural populations sampled of major crop wild progenitors in their habitats of origin (Latitude and Longitude in decimal degrees, WGS84).

Wild progenitor	Modern crop	Country	ID population	Latitude	Longitude
Glycine max subsp. soja	soya	China	P1	40.4543	115.9548
Glycine max subsp. soja	soya	China	P2	40.0181	116.6431
Glycine max subsp. soja	soya	China	P3	39.4228	117.2828
Glycine max subsp. soja	soya	China	P4	39.5383	117.2817
Glycine max subsp. soja	soya	China	P5	37.5567	115.5264
Glycine max subsp. soja	soya	China	P6	38.8639	115.7997
Glycine max subsp. soja	soya	China	P7	39.19	118.3833
Glycine max subsp. soja	soya	China	P8	34.7478	111.1053
Glycine max subsp. soja	soya	China	P9	34.7664	117.1367
Glycine max subsp. soja	soya	China	P10	26.8959	112.5152
Glycine max subsp. soja	soya	China	P11	27.4719	112.1894
Glycine max subsp. soja	soya	China	P12	26.3406	116.2867
Glycine max subsp. soja	soya	China	P13	26.3094	117.4039
Glycine max subsp. soja	soya	China	P14	29.9702	121.4616
Gossypium hirsutum	cotton	Mexico	P1	21.29747	-89.5765
Gossypium hirsutum	cotton	Mexico	P2	21.30112	-89.5563
Gossypium hirsutum	cotton	Mexico	P3	21.30494	-89.5407
Gossypium hirsutum	cotton	Mexico	P4	21.3282	-89.388
Gossypium hirsutum	cotton	Mexico	P5	21.33508	-89.3331
Gossypium hirsutum	cotton	Mexico	P6	21.33123	-89.3601
Gossypium hirsutum	cotton	Mexico	P7	21.01441	-90.3245
Gossypium hirsutum	cotton	Mexico	P8	21.19149	-89.9623
Gossypium hirsutum	cotton	Mexico	P9	21.56142	-87.9989
Gossypium hirsutum	cotton	Mexico	P10	21.56103	-87.9882
Helianthus annuus	sunflower	USA	P1	38.4996	-106.083
Helianthus annuus	sunflower	USA	P2	37.62886	-105.595
Helianthus annuus	sunflower	USA	P3	35.88963	-106.257
Helianthus annuus	sunflower	USA	P4	35.85114	-106.773
Helianthus annuus	sunflower	USA	P5	39.21093	-109.093
Helianthus annuus	sunflower	USA	P6	39.21039	-108.958
Helianthus annuus	sunflower	USA	P7	38.78433	-108.248
Helianthus annuus	sunflower	USA	P8	38.16731	-108.242
Helianthus annuus	sunflower	USA	P9	39.92867	-105.233
Helianthus annuus	sunflower	USA	P10	40.21028	-105.289
Helianthus annuus	sunflower	USA	P11	38.3216	-109.423
Helianthus annuus	sunflower	USA	P12	40.80193	-104.013
Helianthus annuus	sunflower	USA	P13	40.63894	-104.487
Helianthus annuus	sunflower	USA	P14	40.40526	-105.126
Phaseolus vulgaris	bean	Mexico	P1	20.5094	-103.461
Phaseolus vulgaris	bean	Mexico	P2	19.9477	-103.32
Phaseolus vulgaris	bean	Mexico	P3	20.5851	-102.318
Phaseolus vulgaris	bean	Mexico	P4	19.6129	-101.242
Phaseolus vulgaris	bean	Mexico	P5	20.065	-102.613

Table S2 Continued.

Wild progenitor	Modern crop	Country	ID population	Latitude	Longitude
Phaseolus vulgaris	bean	Mexico	P6	19.7537	-101.249
Phaseolus vulgaris	bean	Mexico	P7	20.1263	-101.082
Phaseolus vulgaris	bean	Mexico	P8	23.7802	-105.389
Phaseolus vulgaris	bean	Mexico	P9	18.9971	-99.2362
Phaseolus vulgaris	bean	Mexico	P10	19.0275	-99.2864
Phaseolus vulgaris	bean	Mexico	P11	18.9624	-99.1052
Solanum berthaultii	potato	Bolivia	P1	-17.8503	-65.4215
Solanum berthaultii	potato	Bolivia	P2	-18.1269	-65.2244
Solanum berthaultii	potato	Bolivia	P3	-18.1977	-64.9918
Solanum berthaultii	potato	Bolivia	P4	-18.5628	-65.1277
Solanum berthaultii	potato	Bolivia	P5	-18.9647	-65.1378
Solanum berthaultii	potato	Bolivia	P6	-19.1695	-65.1954
Solanum berthaultii	potato	Bolivia	P7	-19.3279	-65.1868
Solanum berthaultii	potato	Bolivia	P8	-19.5115	-65.246
Solanum berthaultii	potato	Bolivia	P9	-20.589	-65.1303
Solanum berthaultii	potato	Bolivia	P10	-20.9275	-65.1763
Solanum berthaultii	potato	Bolivia	P11	-21.1459	-64.6948
Solanum berthaultii	potato	Bolivia	P12	-17.6584	-65.3925
Zea mays subsp. parviglumis	maize	Mexico	P1	19.91147	-104.173
Zea mays subsp. parviglumis	maize	Mexico	P2	19.93459	-104.006
Zea mays subsp. parviglumis	maize	Mexico	P3	20.6106	-104.214
Zea mays subsp. parviglumis	maize	Mexico	P4	20.60125	-104.187
Zea mays subsp. parviglumis	maize	Mexico	P5	20.57783	-104.387
Zea mays subsp. parviglumis	maize	Mexico	P6	20.161	-101.373
Zea mays subsp. parviglumis	maize	Mexico	P7	20.14123	-101.273
Zea mays subsp. parviglumis	maize	Mexico	P8	20.053	-101.088
Zea mays subsp. parviglumis	maize	Mexico	P9	20.43628	-102.38
Zea mays subsp. parviglumis	maize	Mexico	P10	20.42707	-102.326
Oryza rufipogon	rice	China	P1	23.6775	101.8592
Oryza rufipogon	rice	China	P2	24.1578	117.7994
Oryza rufipogon	rice	China	P3	24.5511	110.4067
Oryza rufipogon	rice	China	P4	23.3742	110.1097
Oryza rufipogon	rice	China	P5	23.5064	109.5044
Oryza rufipogon	rice	China	P6	22.5519	110.0711
Oryza rufipogon	rice	China	P7	21.8592	110.7169
Oryza rufipogon	rice	China	P8	28.0967	116.5444
Oryza rufipogon	rice	China	P9	27.4433	114.1572
Oryza rufipogon	rice	China	P10	19.7867	110.68
Oryza rufipogon	rice	China	P11	19.1103	110.4819
Oryza rufipogon	rice	China	P12	18.7403	110.4103
Hordeum vulgare subsp. spontaneum	barley	Iran	P1	34.97183	46.985
Hordeum vulgare subsp. spontaneum	barley	Iran	P2	34.84422	46.94577
Hordeum vulgare subsp. spontaneum	barley	Iran	P3	35.20603	47.0058
Hordeum vulgare subsp. spontaneum	barley	Iran	P4	35.24953	47.0147
Hordeum vulgare subsp. spontaneum	barley	Iran	P5	35.27703	47.03295

Table S2 Continued.

Wild progenitor	Modern crop	Country	ID population	Latitude	Longitude
Hordeum vulgare subsp. spontaneum	barley	Iran	P6	35.23372	46.96042
Hordeum vulgare subsp. spontaneum	barley	Iran	P7	35.3561	47.01158
Hordeum vulgare subsp. spontaneum	barley	Iran	P8	35.47007	46.95388
Hordeum vulgare subsp. spontaneum	barley	Iran	P9	35.47185	46.98823
Hordeum vulgare subsp. spontaneum	barley	Iran	P10	35.40987	46.83563
Hordeum vulgare subsp. spontaneum	barley	Iran	P11	35.33138	46.64337
Hordeum vulgare subsp. spontaneum	barley	Iran	P12	35.28727	46.98268
Hordeum vulgare subsp. spontaneum	barley	Iran	P13	35.33703	47.15483
Hordeum vulgare subsp. spontaneum	barley	Iran	P14	35.80665	47.15498
Hordeum vulgare subsp. spontaneum	barley	Iran	P15	35.68077	47.11548
Panicum sumatrense	little millet	India	P1	19.21366	81.7141
Panicum sumatrense	little millet	India	P2	18.68659	82.79987
Panicum sumatrense	little millet	India	P3	11.42698	76.7326
Panicum sumatrense	little millet	India	P4	12.02641	77.11298
Panicum sumatrense	little millet	India	P5	12.43671	76.73347
Panicum sumatrense	little millet	India	P6	12.28398	76.69049
Panicum sumatrense	little millet	India	P7	12.06687	76.10879
Panicum sumatrense	little millet	India	P8	11.97878	76.05441
Panicum sumatrense	little millet	India	P9	16.25234	80.27971
Panicum sumatrense	little millet	India	P10	17.44267	78.33831
Panicum sumatrense	little millet	India	P11	28.49213	80.65044
Panicum sumatrense	little millet	India	P12	25.33839	90.56481
Panicum sumatrense	little millet	India	P13	25.26707	90.72977
Panicum sumatrense	little millet	India	P14	24.65741	79.84126
Panicum sumatrense	little millet	India	P15	31.78028	76.99946
Triticum dicoccoides	wheat	Israel	P1	32.6003	35.0614
Triticum dicoccoides	wheat	Israel	P2	32.605	35.0711
Triticum dicoccoides	wheat	Israel	P3	32.9431	35.5717
Triticum dicoccoides	wheat	Israel	P4	32.9344	35.5756
Triticum dicoccoides	wheat	Israel	P5	32.9217	35.5972
Triticum dicoccoides	wheat	Israel	P6	32.905	35.5972
Triticum dicoccoides	wheat	Israel	P7	32.93	35.6894
Triticum dicoccoides	wheat	Israel	P8	32.9819	35.7506
Triticum dicoccoides	wheat	Israel	P9	32.8967	35.77
Triticum dicoccoides	wheat	Israel	P10	32.8642	35.7794
Triticum dicoccoides	wheat	Israel	P11	32.8131	35.7172
Triticum dicoccoides	wheat	Israel	P12	32.7689	35.7119

Table S3 Primary productivity, climate, and soil chemistry by ecoregions of wild populations clustered in the PCA (Figure 2).

Ecoregion	Mean NDVI			AI			MAT (°C)			MAP (mm)			pH			Sand (%)			OC (%)			PO_4^- (mg kg^{-1})			NH_4^+ (mg kg^{-1})			CE (µs cm^{-1})		
	Mean	SD		Mean	SD		Mean	SD		Mean	SD		Mean	SD		Mean	SD		Mean	SD		Mean	SD		Mean	SD		Mean	SD	
Deserts	0.28	0.1	b	0.28	0.1	c	13.7	3.8	d	483.7	102.2	c	8.0	0.4	a	36.2	16.6	c	1.4	1.2	b	6.9	3.5	b	5.9	3.7	b	172.1	217.2	b
Coastal xeric shrublands	0.46	0.1	a	0.38	0.0	bc	25.9	0.2	a	712.6	48.6	bc	8.3	0.2	a	88.0	3.0	a	1.0	0.2	ab	9.0	2.0	ab	2.4	0.8	b	1044.7	679.9	a
Temperate dry forest and shrublands	0.48	0.1	a	0.44	0.2	b	17.4	5.4	c	761.6	311.1	b	7.0	1.0	b	60.6	18.1	b	1.6	1.3	ab	10.3	6.5	a	8.0	5.7	b	132.8	150.6	b
Tropical seasonal forest and savanna	0.55	0.1	a	0.97	0.4	a	20.3	3.5	b	1465.4	540.8	a	6.2	1.1	c	45.5	20.6	c	2.2	1.6	a	6.5	3.3	b	15.2	12.4	a	121.0	94.0	b

Letters indicate significant differences among ecoregions (significance level adjusted with Bonferroni correction to account for multi-group comparisons, α = 0.05)

Table S4 Soil fertility (mg kg^{-1}) by ecoregions of wild populations clustered in the PCA (Figure 2).

Ecoregion	Ca			K			Mg			B			Cu			Mn			Fe			Zn		
	Mean	SD		Mean	SD		Mean	SD		Mean	SD		Mean	SD		Mean	SD		Mean	SD		Mean	SD	
Deserts	29489	23251	b	7806	4756	a	9677	4505	a	15.2	13.1	a	40.2	13.3	a	967	618	a	42638	15448	a	93.6	36.3	b
Coastal xeric shrublands	199045	33975	a	239	63	c	3298	684	b	0.0	0.0	b	0.9	0.5	c	42	7	c	313	137	c	2.2	0.5	c
Temperate dry forest and shrublands	9997	10163	c	5050	4756	b	3152	2501	b	2.8	5.0	b	16.9	10.0	b	468	287	bc	20550	12456	b	53.0	28.2	c
Tropical seasonal forest and savanna	7146	9055	c	3903	4017	bc	4221	2711	b	6.8	8.0	b	38.5	14.4	a	593	564	b	48732	24861	a	135.5	111.2	a

Letters indicate significant differences among ecoregions (significance level adjusted with Bonferroni correction to account for multi-group comparisons, α = 0.05.
